# Immunosenescence in Aging and Cancer: Molecular Mechanisms, Tumor Microenvironment Remodeling, and Therapeutic Implications

**DOI:** 10.3390/biom16101487

**Published:** 2026-10-13

**Authors:** Yusra Zarlashat, Syed Salman Shah, Seerat-Uz-Zahra, Rosa Di Lorenzo, Anna Picca

**Affiliations:** 1 Department of Biochemistry, Government College University Faisalabad, Faisalabad 38000, Pakistan; yusrazarlashat@gcuf.edu.pk; 2 Department of Biotechnology and Genetic Engineering, Hazara University, Mansehra 21300, Pakistan; sshah7438@gmail.com; 3 Gomal Center of Biotechnology and Biochemistry (GCBB), Gomal University, Dera Ismail Khan 29050, Pakistan; seeratzahra974@gmail.com; 4 Department of Biosciences Biotechnologies and Environment, University of Bari Aldo Moro, 70125 Bari, Italy; rosa.dilorenzo@uniba.it; 5 Department of Medicine and Surgery, LUM University, 70010 Casamassima, Italy; 6 Fondazione Policlinico Universitario “A. Gemelli” IRCCS, 00168 Rome, Italy

**Keywords:** Immunosenescence, mitochondrial dysfunction, tumor microenvironment, cancer immunotherapy, inflammaging, aging immune system, immune checkpoint inhibitors, mitochondrial quality control, chronic inflammation

## Abstract

Immunosenescence is the decline in immune function with age, which influences cancer progression and treatment outcomes. This process arises from multiple, interconnected molecular and cellular alterations. Among these, mitochondrial dysfunction in aged immune cells may contribute to impaired immune function through altered metabolism, redox imbalance, and inflammatory signaling. This review discusses mitochondrial alterations alongside other age-related mechanisms that shape immune cell function and may promote cancer development. We examine how DNA damage, telomere shortening, epigenetic changes, altered proteostasis, and mitochondrial dysfunction contribute to immune aging and potentially compromise antitumor immunity. The aged tumor microenvironment is characterized by increased immunosuppressive cell populations, persistent inflammatory signaling, and metabolic alterations that can influence tumor development and therapeutic responses. We also examine how aging affects response to current cancer immunotherapies. While some treatments show comparable efficacy in older and younger patients, others may be less effective in the context of age-related immune dysfunction. Older patients may also experience distinct toxicity profiles that require careful clinical management. Understanding how mitochondrial alterations interact with other mechanisms of aging to shape immunosenescence may help identify therapeutic strategies for cancer in older adults. Future approaches may combine interventions targeting mitochondrial fitness, chronic inflammation, and cellular senescence. However, clinical studies specifically designed to include older populations are needed to establish the relevance of these mechanisms in cancer and to guide age-specific therapeutic strategies.

## 1. Introduction

Aging is an irreversible, complex biological process characterized by a progressive, steep decline at the organismal level and in immune competence, a phenomenon known as immunosenescence. This decline drastically increases organismal susceptibility to chronic diseases and contributes to cancer development in older individuals. At the macro-structural level, this decay is underpinned by progressive thymic atrophy coupled with impaired hematopoietic activity within the bone marrow niche. Together, these central lymphoid tissue alterations skew stem cell differentiation pathways away from lymphoid lineages and heavily toward the production of myeloid cells. This phenomenon, referred to as myeloid skewing, profoundly alters the composition of circulating immune cell populations, generating an imbalance that impairs adaptive immunity while fostering a pro-inflammatory state [1]. On the other hand, cellular senescence is a stable state of cell cycle arrest accompanied by characteristic molecular and secretory changes, including the senescence-associated secretory phenotype (SASP), and occurs in both immune and non-immune cells. SASP is the collection of inflammatory cytokines, chemokines, growth factors, and proteases secreted by senescent cells. Conversely, immune cell exhaustion refers to a state of progressive functional impairment that develops primarily after persistent antigenic stimulation, particularly in T cells, and is characterized by reduced effector function and altered expression of inhibitory receptors [2,3].

At the cellular level, the aging of immune cells is characterized by severe bioenergetic compromises, notably defective energy metabolism and extensive mitochondrial dysfunction. As mitochondria lose their membrane potential and structural integrity, they become “leaky”, releasing mitochondrial DNA (mtDNA) and reactive oxygen species (ROS) into the cell cytoplasm. These intracellular danger signals trigger the permanent activation of inflammatory pathways, driving the secretion of chemokines and cytokines known as SASP. Collectively, these continuous cellular alterations establish systemic, sterile, chronic low-grade inflammation, or inflammaging, as a defining phenotypic manifestation of immune aging [4]. This metabolic and inflammatory breakdown shapes the aging tumor microenvironment (TME). The TME refers to a complex network of tumor cells, immune cells, stromal cells, and soluble factors interacting with tumor cells. Aging cells within the TME rely on this localized SASP and persistent inflammatory signaling to remodel the extracellular matrix and compromise tissue architecture, directly contributing to accelerated tumor growth and metastatic spread [5]. Furthermore, the capacity of infiltrating immune cells to mount an effective antitumor response is intrinsically tied to their individual mitochondrial fitness. When entering the immunosuppressive TME, metabolically compromised immune cells develop cellular senescence or functional exhaustion, characterized by impaired proliferation, reduced effector activity, and metabolic dysfunction [2,3]. This exhaustion blunts their cytotoxic potential and prevents them from effectively neutralizing malignant cells. Consequently, this metabolic failure establishes a direct axis between mitochondrial quality control (MQC) homeostasis, the balance of processes that maintain mitochondrial integrity and function, metabolic reprogramming, and the ultimate clinical efficacy of modern cancer immunotherapies [6,7].

In recent years, tumor immunotherapy has revolutionized oncological treatment and now encompasses several advanced modalities, including immune checkpoint inhibitors, molecularly targeted therapies, and adoptive cell transfer (ACT), such as chimeric antigen receptor (CAR)-T and CAR-natural killer (NK) cell therapies, tumor-infiltrating lymphocytes (TILs), dendritic cell (DC) vaccines, and cytokine-induced killer (CIK) cell therapy, as well as cancer vaccines and cytokine-based regimens [8]. Given the progressive aging of the global population and the marked, corresponding increase in cancer incidence with advancing age, clarifying the exact molecular intersection between aging and cancer research has become a priority. However, reliably tracking and quantifying this dynamic shift remains difficult due to a lack of validated biomarkers [9].

In this review, we address this critical gap by proposing three strict criteria that a true immunosenescence marker must fulfill to reliably reflect this process: (1) a progressive onset during aging; (2) a causal relationship with immunological decline, whereby an increase or worsening of the marker directly drives the progression of immune dysfunction; and (3) a capacity for reversibility or modulation upon targeted therapeutic intervention. We systematically assess some of the hallmarks of aging, including genomic instability, telomere attrition, epigenetic alterations, mitochondrial dysfunction, loss of proteostasis, altered intercellular communication, and chronic inflammation, against these proposed criteria to determine their relevance as genuine, actionable hallmarks of immunosenescence. For instance, although stem cell depletion is widely regarded as a primary hallmark of organismal aging, we hypothesize that downstream cellular defects like mitochondrial dysfunction and epigenetic alterations more accurately reflect the true immune aging process. This is because they directly drive immediate functional impairments, such as defective autophagy, enhanced SASP expression, and chronic inflammatory polarization. Ultimately, these highly interconnected mechanisms do not act in isolation; their synergistic effects establish a systemic state of immunosenescence that blunts cytotoxic responses, fuels persistent low-grade inflammation, and compromises immune surveillance, directly driving cancer initiation, progression, and therapeutic resistance. Recognizing this therapeutic potential, cutting-edge interventions are now pointing toward breaking this cycle through advanced aging clocks, cellular and mitochondrial reprogramming, the targeted clearance of senescent cells via senolytics, and innovative techniques for thymic rejuvenation [10].

## 2. Molecular and Cellular Hallmarks of Immunosenescence

### 2.1. Genomic Instability

Among the primary mechanisms of biological decay, genomic instability and epigenetic alterations represent primary upstream instigators of the immunosenescent phenotype. During aging, circulating lymphocytes show a progressive accumulation of DNA damage alongside a profound reshaping of their epigenetic architecture. Several reliable biomarkers of this nuclear-genomic decay have been identified in leukocytes, including standard micronuclei (MNs) and centromere-positive micronuclei (MNC^+^). MNs are small extranuclear bodies containing chromosomal fragments or whole chromosomes excluded from the main nucleus, while MNC^+^ specifically contain centromeric DNA [11]. A classic manifestation of this epigenetic drift is the hypomethylation of long interspersed element-1 (LINE-1) retrotransposons and the structural destabilization of ALU-J/sx elements. Crucially, while LINE-1 hypomethylation correlates with advancing age in human lymphocytes, it is not significantly associated with structural MNC^+^ levels and exhibits substantial geographical variability among aging populations [11]. Concurrently, the progressive exhaustion of endogenous DNA repair pathways, such as double-strand break repair and mismatch repair, fails to counteract this continuous genomic insult, forcing aged immune cells into a state of permanent arrest or senescent polarization [12]. For instance, the deregulation of mismatch repair genes leads to microsatellite instability, a phenomenon closely linked to the age-dependent depletion of MutL heterodimers observed in cloned T cells in vitro. This chronic activation of the DNA damage response (DDR) directly fuels downstream intracellular crises, most notably extensive mitochondrial dysfunction [13]. Age-related oxidative stress may also promote the horizontal translocation of mitochondrial DNA (mtDNA) portions into the nucleus, where it preferentially integrates close to telomeric regions in T cells, resulting in severe genetic alterations, accelerated MN formation, and a decreased capacity for cellular proliferation [13]. Another hallmark of this interconnected breakdown is the increased expression of the marker CD63 following the loss of nuclear Lamina A/C, a process driven by persistent DNA damage and nucleotide excision repair activation. When the integrity of both the nuclear and mitochondrial genomes is compromised, the cell loses control over its bioenergetic homeostasis. Dysfunctional mitochondria show an altered membrane potential, severe ATP insufficiency, and a marked overproduction of ROS. Ultimately, this combined breakdown, characterized by chronic DNA damage, LINE-1 retrotransposition stress, and MQC failure, acts as a critical driver of the SASP, likely converting aged immune cells into active engines of systemic, sterile inflammation [2,3,4].

### 2.2. Telomere Attrition

Immunosenescence is closely intertwined with telomere attrition, a hallmark process defined by progressive telomeric shortening and a concomitant decline in telomerase activity [14]. Leukocyte telomere length (LTL), the length of telomeres at the ends of chromosomes in circulating leukocytes, has emerged as one of the most extensively investigated biomarkers of aging. This measure, indeed, reflects cellular replicative history and biological aging, thus serving as a reliable surrogate indicator of both cellular senescence and systemic biological age [15]. In clinical and research settings, LTL is routinely quantified using several established methodologies, including flow-fluorescence in situ hybridization, quantitative polymerase chain reaction, and terminal restriction fragment (TRF) analysis [16]. Notably, biological aging does not always align with chronological aging; for instance, accelerated LTL attrition serves as a predictor of cognitive aging even in otherwise healthy young individuals, whereas marked telomeric erosion is more frequently documented in older cohorts [14]. To circumvent the technical limitations of direct measurement, several circulating biomarkers have been identified to indirectly predict LTL dynamics, including Fetuin-A [17] and specific metabolic profiles [18]. Furthermore, several genetic, environmental, and lifestyle-related variables actively accelerate telomere shortening, though their quantified contributions often vary depending on the specific detection approaches employed [19]. Beyond its association with chronological aging, LTL attrition is independently driven by chronic infectious diseases, particularly viral infections such as human immunodeficiency virus (HIV), cytomegalovirus, herpes simplex virus type 1, and human herpesvirus 6 [20]. Mechanistically, HIV infection directly triggers telomeric erosion and subsequent T-cell senescence by actively suppressing telomerase activity alongside key nuclear components, including telomeric repeat binding factor 2, ataxia telangiectasia mutated (ATM) kinase, and topoisomerase I and II alpha kinetics. These multi-axial enzymatic inhibitions culminate in the critical accumulation of unrepaired DNA damage. This genotoxic stress is evidenced by elevated expression of the DNA damage marker γ-H2A family member X, the formation of dysfunctional telomere-induced foci, and the stabilization of the cytotoxic topoisomerase cleavage complex [21].

### 2.3. Epigenetic Alterations

**Figure 1:**
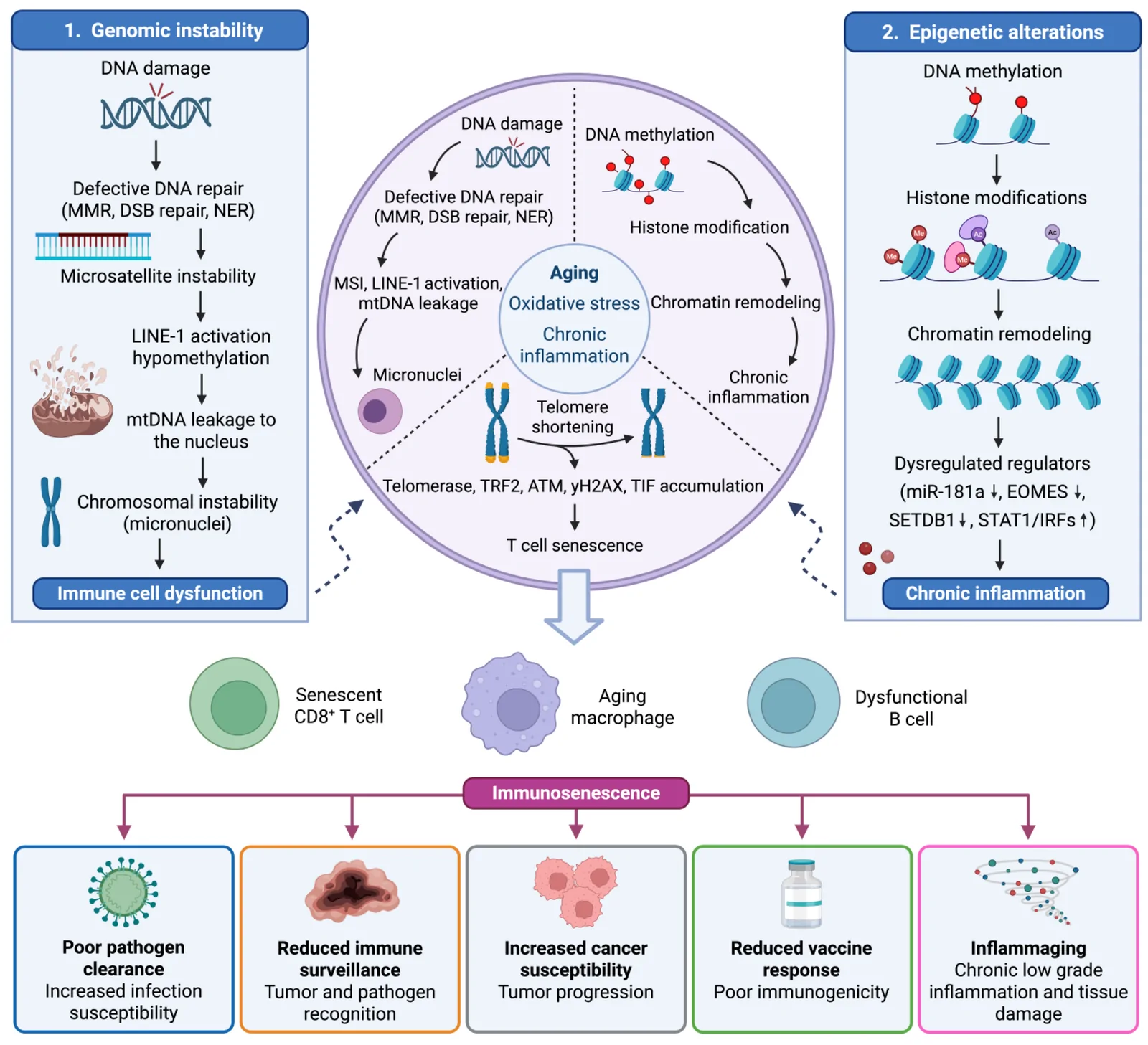
Molecular mechanisms by which genomic instability, telomere attrition, and epigenetic alterations trigger immunosenescence. Age-associated oxidative stress and chronic inflammation promote genomic instability through DNA damage, defective DNA repair, MSI, LINE-1 activation, mtDNA leakage, and micronuclei formation. Telomere attrition, characterized by telomere shortening, reduced telomerase activity, and activation of persistent DNA damage responses, induces T-cell senescence and impaired proliferative capacity. Epigenetic alterations, including abnormal DNAm, histone modifications, chromatin remodeling, and dysregulation of key epigenetic regulators, further promote chronic inflammation and immune dysfunction. Together, these interconnected processes impair the function of T cells, B cells, and macrophages, leading to immunosenescence, which is associated with reduced immune surveillance, inflammaging, impaired pathogen clearance, increased cancer susceptibility, and reduced vaccine responses. Abbreviations: ATM, ataxia-telangiectasia mutated; CD8^+^, cluster of differentiation 8-positive; DSB, double-strand break; EOMES, eomesodermin; γH2AX, phosphorylated histone H2AX; IRFs, interferon regulatory factors; LINE-1, long interspersed nuclear element-1; miR-181, microRNA-181; MMR, mismatch repair; MSI, microsatellite instability; mtDNA, mitochondrial DNA; NER, nucleotide excision repair; SETDB1, SET domain bifurcated histone lysine methyltransferase 1; STAT1, signal transducer and activator of transcription 1; T cell, T lymphocyte; TIF, telomere dysfunction-induced foci; TRF2, telomeric repeat-containing factor 2. Created with BioRender.com accessed on 5 September 2026.

Biological aging is accompanied by profound epigenetic remodeling that contributes to the initiation and progression of immunosenescence. Among the earliest and most widely adopted epigenetic tools, first-generation aging clocks estimate biological age based on DNA methylation (DNAm) patterns at 353 CpG sites across heterogeneous tissues and isolated cell subsets, such as monocytes, neutrophils, CD4^+^ T cells, and immortalized B cells [22]. However, these pioneer epigenetic metrics, including the Horvath, Hannum, Blood, and PhenoAge clocks, are frequently confounded by age-related shifts in immune cell composition, particularly the changing differentiation states of CD8^+^ T cells within peripheral blood mononuclear cells [23]. To circumvent this limitation, the innovative IntrinClock provides a more robust and consistent age prediction across distinct immune subsets by selectively targeting CpG regions that remain stable throughout CD8^+^ T-cell development. These deep epigenetic alterations are closely intertwined with inflammaging, establishing a reciprocal feedback loop where epigenetic modifications both promote and are reinforced by persistent systemic inflammation. These epigenetic adjustments drive immune decay through both tissue-specific and systemic axes; for instance, high-resolution ATAC-seq analyses have revealed that the transcription factor eomesodermin, a key member of the T-box family, epigenetically regulates the phenotypic conversion of resting CD8^+^ T cells into age-associated, granzyme K (GZMK)-expressing CD8^+^ T (Taa) cells. GZMK-expressing CD8^+^ T cells are a subset of CD8^+^ T cells that produce granzyme K and are associated with inflammatory and age-related immune responses. These Taa cells progressively accumulate in both older humans and mice, upregulate the surface homing integrin CD49d, display an enhanced capacity to migrate into peripheral tissues, and directly fuel systemic inflammation via sustained GZMK secretion [24]. Concurrently, the age-associated decline of miR-181a disrupts the finely tuned miR-181a/sirtuin 1 (SIRT1) axis. Under physiological conditions, SIRT1 acts as an epigenetic repressor of histone genes by binding to histone promoters and reducing histone acetylation; thus, a loss of miR-181a leads to defective histone expression, delayed cell cycle progression, and increased replication stress, compromising T-cell activity and inducing a marked overproduction of inflammatory cytokines in both miR-181a/b1-deficient murine T cells and naïve T cells from older individuals [25]. Notably, targeted treatment with SIRT1 inhibitors successfully rescues this phenotype, dampening aberrant inflammatory cytokine production in mice carrying miR-181a-deficient T cells [26]. Furthermore, in senescent CD8^+^ CD28-T-cell subpopulations, a distinct reduction in histone H2AX expression limits the biological availability of miR-24 targets, selectively blunting the cell’s capacity to trigger an effective DDR [27]. Higher-order chromatin architecture remodeling also plays a critical role in immune decay, as age-dependent three-dimensional chromatin reorganization within bone marrow progenitor B cells markedly decreases structural connectivity within topologically associated domains, resulting in the coordinated downregulation of critical gene networks essential for B-cell growth and consequently defective B lymphopoiesis [28]. Epigenetic instability at the tissue level further amplifies inflammation-induced immunosenescence through distinct cell-autonomous pathways. For example, hyperglycemia-induced downregulation of the SET domain bifurcated histone lysine methyltransferase (SETDB1) in macrophages relieves transcriptional repression at the LINE-1 promoter, regulating senescence-like phenotypical changes and M1 pro-inflammatory polarization that aggravate periodontal inflammaging in diabetic conditions. Notably, metformin treatment was shown to restore SETDB1 activity and alleviate this hyperglycemia-induced periodontal inflammaging in preclinical models [29]. This cell-intrinsic inflammatory drive is mirrored in non-hematopoietic niches, where aged murine intestinal stem cells show increased chromatin accessibility at inflammation-related genetic loci, actively propagating tissue-level inflammation through mechanisms that remain to be fully elucidated [30]. Similarly, interferon signaling pathways serve as major drivers of age-associated chronic inflammation; older individuals display a marked upregulation of signal transducer and activator of transcription 1 (STAT1) and interferon regulatory factors (IRFs) [31]. In murine kidney and liver models, widespread age-related increases in chromatin accessibility dictate the abnormal expression of these transcription factors, a pathological state that can be partially attenuated by long-term dietary restriction through a targeted reduction in chromatin accessibility at STAT1- and IRF-associated regulatory regions, offering a safe, non-invasive intervention to mitigate systemic inflammaging [32]. Conversely, systemic inflammation acts as a potent accelerator of the epigenetic clock, creating a self-sustaining pathogenic cycle. In human skin, chronic ultraviolet light exposure induces persistent epidermal inflammation that promotes accelerated epigenetic aging marked by stable DNAm alterations that persist for decades [33] across specific, though not all, sun-exposed anatomical regions. Pathogen-driven chronic inflammation similarly accelerates the epigenetic clock; for instance, direct-acting antivirals can successfully reverse the epigenetic age acceleration associated with hepatitis C virus (HCV) infection following a sustained virologic response (SVR) [34]. However, because these DNAm analyses were historically performed on peripheral blood mononuclear cells using the first-generation Horvath clock rather than directly on hepatocytes, it remains difficult to decouple whether this accelerated epigenetic aging is driven natively by intrahepatic HCV replication or by systemic chronic inflammation [35]. This pathogen-mediated epigenetic acceleration is strongly linked to long-term exposure to vulnerable, hyper-inflammatory environments; population-scale demographic data show that accelerated epigenetic aging is prevalent among residents of leprosy-endemic areas regardless of their individual infection status [36], indicating distinct geographical variability in epigenetic age acceleration profiles across both leprosy and HCV cohorts [36]. In the clinical setting, these advanced epigenetic biomarkers are rapidly emerging as invaluable translational tools for quantifying immunosenescence and systemic low-grade inflammation in older cohorts [3]. Notably, the Epigenetic Inflammation Score, derived from specific CpG methylation signatures, significantly outperforms conventional C-reactive protein measurements in detecting subtle, low-grade inflammation in older people [37]. Additionally, peripheral blood DNAm profiling accurately predicts pathological immune cell infiltration in chronic neurodegenerative and vascular disorders, such as atherosclerosis and Alzheimer’s disease [38], highlighting the vast clinical potential of epigenetic modifications as biomarkers for disease diagnosis, prognosis, and therapeutic monitoring. While many age- and inflammation-related epigenetic changes follow predictable canonical rules, such as DNAm-mediated gene silencing and histone acetylation-mediated transcriptional activation, recent studies have uncovered notable exceptions [29] that reveal tissue-specific regulatory nuances. For instance, the age-associated hypomethylation of the inflammation-related gene CXCL10 increases its expression. Surprisingly, elevated CXCL10 expression is linked to improved spatial memory in older adults carrying the rs56061981 genetic variant, which eliminates the CpG-136 site and further enhances CXCL10 transcription [39]. Similarly, histone H3 lysine 27 acetylation (H3K27ac), traditionally characterized as an activating transcriptional mark, is paradoxically reduced at pro-inflammatory gene bodies that become highly expressed in the aging brain, a disruptive phenotype that can be suppressed via treatment with histone deacetylase inhibitors (HDACis) [40], indicating a tissue-specific regulatory function for H3K27ac during aging. Beyond their baseline anti-inflammatory efficacy, HDACis possess significant therapeutic value in oncology by preventing chimeric antigen receptor T-cell (CAR-T cell) dysfunction and exhaustion; they achieve this by stimulating the wingless-related integration site (Wnt)/β-catenin pathway through the upregulated expression of the essential transcription factors LEF1A and TCF4. Collectively, these multi-axial findings indicate the intricate, synergistic crosstalk linking genomic instability, telomere attrition, and epigenetic alterations to the systemic manifestation of immunosenescence, as summarized in Figure 1.

### 2.4. Loss of Proteostasis

Proteostasis refers to the dynamic balance of cellular processes governing protein synthesis, folding, trafficking, repair, and degradation. Aging progressively compromises this cellular protein homeostasis, resulting in a systemic decline in proteostatic capacity that contributes significantly to immunosenescence [41]. Current evidence indicates that proteostasis dysfunction within the aging immune system is primarily driven by two interconnected pathways: impaired autophagy and abnormal unfolded protein response (UPR) signaling, particularly within microglia and macrophage lineages [42]. Impairment of mitophagy in macrophages promotes a phenotypic shift toward the pro-inflammatory M1 state under periodontitis-associated inflammatory conditions, whereas the restoration of this mitophagy capacity represents a promising therapeutic strategy [43]. For instance, stimulation with resveratrol enhances PINK1-mediated mitophagy, reduces mitochondrial ROS production, and promotes a functional transition from the M1 to the anti-inflammatory M2 phenotype in macrophages [43]. Similarly, pharmacological manipulation via rapamycin treatment effectively rejuvenates the host regenerative potential; in aged rats, rapamycin promotes the degradation of biodegrading poly(glycerol sebacate)-polycaprolactone vascular grafts, facilitating their in situ infiltration and gradual development into functional blood vessel-like structures during vascular remodeling [44]. An analogous age-dependent impairment of autophagy is observed in microglia, the resident mononuclear macrophages of the central nervous system, where it actively propagates neuroinflammation and cognitive decline [45]. This neuroprotective degradation is particularly relevant in peri-operative neurocognitive disorders, a cluster of cognitive deficiencies induced by surgery and anesthesia that occur more commonly in older people and are largely driven by microglia-mediated neuroinflammation coupled with defective autophagy [46]. Several targeted interventions have shown promise in reversing this cognitive decay by restoring microglial autophagy. Administration of β-caryophyllene, a naturally occurring terpene that selectively activates cannabinoid receptor 2, restores autophagic ability and prevents hippocampal microglia from releasing the pro-inflammatory cytokines interleukin-1 (IL-1) and IL-6 in aged mice [47]. Conversely, traumatic brain injury (TBI), which carries a worse prognosis and a significantly greater mortality rate in older individuals, exacerbates this baseline age-related decline by reducing microglial autophagy while aberrantly increasing phagocytosis, oxidative stress, and pro-inflammatory phenotypic polarization, ultimately aggravating neurodegeneration and neuroinflammation [48]. Remarkably, treatment with trehalose, an autophagy activator acting upstream of the lysosomal system, restrains this excessive microglial phagocytosis, thereby recovering both motor and cognitive abilities in aged post-TBI mice [49]. Another critical homeostatic mechanism involves the IL-34–colony-stimulating factor 1 receptor–extracellular signal-regulated kinase/AMP-activated protein kinase (ERK/AMPK) signaling axis, which promotes the differentiation of an autophagy-dependent age-acquired microglia (ADAM) subpopulation that mitigates neuroinflammation. ADAM is, indeed, an age-acquired microglial subpopulation characterized by increased autophagy and age-dependent functional changes. Although ADAM abundance progressively declines during chronological aging, exogenous IL-34 supplementation successfully restores their population, highlighting its therapeutic value for age-related neurodegenerative disorders [50]. In addition to defective autophagy, alterations in UPR signaling cascades heavily contribute to proteostasis failure in aged myeloid cells across multiple tissues, ultimately resulting in disrupted protein homeostasis. In aged microglia, aberrant or sustained UPR activation severely impairs proteostasis, whereas supplementation with the polyphenol mix NT-020 successfully attenuates this sustained UPR signaling and improves overall protein homeostasis, even though the precise molecular mechanisms driving microglial aging remain poorly understood [51]. Conversely, tissue-resident CX_3_CR1^+^ macrophages isolated from aged mice recovering from influenza A virus-induced pneumonia show a marked downregulation of core UPR components alongside the elevated expression of genes associated with severe proteostatic stress [52]. These apparently contradictory findings likely reflect tissue-specific differences, species-dependent variability, or the confounding influence of viral infection, emphasizing the critical need for further mechanistic research using standardized experimental models. Age-related proteostatic failure also directly interferes with adaptive immune responses by compromising classic antigen presentation pathways. Endosomal proteostasis and major histocompatibility complex (MHC) class II antigen presentation are disrupted in dendritic cells (DCs) by chronic oxidative stress, which induces aberrant protein modifications, such as glycation, carbonylation, and lipoxidation, culminating in the pathogenic accumulation of insoluble protein aggregates within late endosomal compartments [53]. Finally, age-dependent alterations have been extensively described within the proteasome system, where standard proteasomes and immunoproteasomes differ in the composition of their catalytic subunits. In aged rat hepatocytes, immunoproteasome-specific subunits partially replace standard subunits to generate intermediate-proteasome complexes characterized by reduced hydrolytic activity [54]. Because immunoproteasomes play a crucial role in generating antigenic peptides for MHC class I presentation, these age-associated proteasomal alterations blunt antigen processing and presentation, thereby directly linking cell-autonomous proteostatic dysfunction to impaired adaptive immunity and altered leukocyte ratios [55].

### 2.5. Mitochondrial Dysfunction

Mitochondria are double-membraned organelles of all eukaryotic cells; while primarily known as ATP producers, they also serve as critical signaling hubs regulating calcium homeostasis, apoptosis, and immune cell activation. Mitochondrial dysfunction is among the main contributors to the aging process. Age-related mitochondrial genetic variations have been associated with human aging and longevity. In a study of individuals older than 100 years, inherited mtDNA haplogroups differed from those of younger control counterparts, with haplogroup J being more frequent among male centenarians, suggesting that inherited mitochondrial variations contribute to successful aging and longevity [56]. Similarly, targeted sequencing of approximately 660 nuclear-encoded mitochondrial genes in 496 centenarians and 572 controls identified rare variants in genes involved in several mitochondrial pathways associated with exceptional longevity, including those involved in mitochondrial translation and mitochondrial dynamics [57]. More recently, whole-genome sequencing of approximately 750,000 individuals showed that mtDNA heteroplasmy increases with age in human blood and identified nuclear genetic loci associated with age-related mitochondrial variant accumulation, providing further evidence that mitochondrial genetic and cellular changes are linked to aging [58].

**Figure 2:**
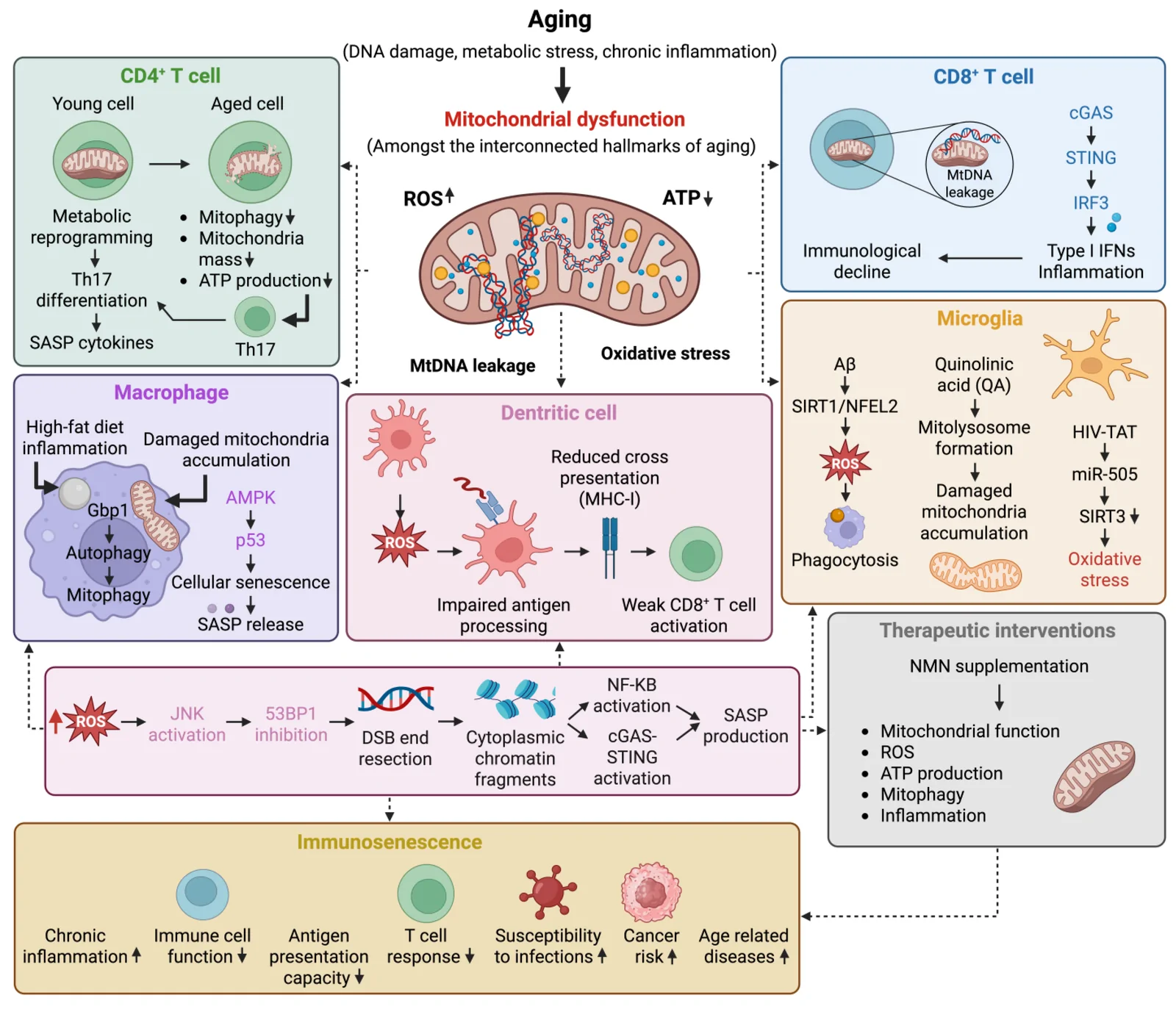
The contribution of mitochondrial dysfunction to immunosenescence. Mitochondrial dysfunction regulates immune cell-specific immunosenescence during aging. Aging-related mitochondrial decline, characterized by impaired mitophagy, reduced ATP production, increased ROS generation, and mtDNA leakage, disrupts the function of CD4^+^ and CD8^+^ T cells, macrophages, DCs, and microglia through distinct molecular pathways. These changes can trigger chronic inflammation, SASP production, impaired antigen presentation, and reduced immune responses, ultimately leading to immunosenescence. Potential therapeutic interventions, including NMN supplementation, have been shown to potentially alleviate mitochondrial dysfunction and inflammatory responses. *Abbreviations***:** Aβ, amyloid-beta; AMPK, AMP-activated protein kinase; ATP, adenosine triphosphate; CD4^+^, cluster of differentiation 4-positive; CD8^+^, cluster of differentiation 8-positive; cGAS, cyclic GMP-AMP synthase; DSB, double-strand break; GBP1, guanylate-binding protein 1; HIV-TAT, human immunodeficiency virus transactivator of transcription; IFNs, interferons; IRF3, interferon regulatory factor 3; JNK, c-jun N-terminal kinase; MHC-I, major histocompatibility complex class I; miR-505, microRNA-505; mtDNA, mitochondrial DNA; NF-κB, nuclear factor kappa B; NMN, nicotinamide mononucleotide; p53, tumor protein p53; ROS, reactive oxygen species; NFE2L2, nuclear factor erythroid 2-like 2; SASP, senescence-associated secretory phenotype; SIRT1, sirtuin 1; STING, stimulator of interferon genes; T cell, T lymphocyte; Th17, T helper 17; 53BP1, p53-binding protein 1. Created with BioRender.com accessed on 5 September 2026.

To maintain cellular homeostasis, mitochondrial number, quality, and shape are tightly regulated by interconnected MQC systems [59]. These systems include mitophagy, a selective form of autophagy that removes damaged organelles through the canonical PINK1/Parkin pathway, and dynamic cycles of fusion, mediated by mitofusin 1 and 2 and optic atrophy 1, and fission, driven by dynamin-related protein 1 [59]. Furthermore, protein homeostasis within the organelle is monitored by the mitochondrial unfolded protein response (UPRmt), which facilitates the refolding or degradation of misfolded intra-mitochondrial proteins [60]. This adaptive stress pathway plays a particularly protective role in preserving regulatory T cell function during immune aging [61]. Concurrently, mitochondrial biogenesis is governed by key transcription factors, including peroxisome proliferator-activated receptor gamma coactivator 1-alpha (PGC-1α) and nuclear respiratory factor 2 (NRF-2), to meet cellular energy demands [62]. Under conditions where MQC systems fail, low-quality mitochondria can release ROS and mtDNA into the cytoplasm; these components act as damage-associated molecular patterns (DAMPs) that activate the innate immune response via the NLR family pyrin domain containing 3 inflammasome and the cyclic GMP-AMP synthase (cGAS)-stimulator of interferon genes (STING) pathway [63]. Consequently, the age-related failure of MQC networks serves as a central driver of immunosenescence, shifting immune cell fate through metabolic reprogramming (Figure 2).

While naive and memory T cells rely on oxidative phosphorylation for long-term survival, effector T cells depend heavily on glycolysis, requiring precise metabolic transitions during differentiation [64]. Aging severely compromises mitochondrial fitness across various immune cell subsets, establishing a self-reinforcing loop of chronic inflammation and functional decline. Senescent T cells, which accumulate progressively with age, exhibit a marked reduction in mitochondrial mass and impaired oxidative metabolism [65]. Remarkably, the experimental transfer of functional mitochondria into senescent CD4^+^ effector memory T cells re-expressing cluster of differentiation 45 receptor type A (CD45RA, T_EMRA_) restores aerobic respiration and controls in vitro infections [66]. Conversely, aged memory CD4^+^ T cells display an unexpected increase in mitochondrial mass [67]. Although this adaptation elevates maximum oxygen consumption, it triggers excessive ROS generation that drives T helper 17 (Th17) polarization and fuels SASP-mediated inflammation via the enhanced production of pro-inflammatory cytokines, including interferon-γ (IFN-γ), IL-4, IL-6, IL-8, and IL-9 [64]. This elevated oxidative stress compromises other immune populations; in dendritic cells (DCs), excessive ROS production impairs antigen processing and blunts CD8^+^ T-cell priming and cross-presentation independently of total mitochondrial mass [68]. Furthermore, aging lymphocytes display an increased susceptibility to mitochondrial permeability transition (PT), causing mitochondrial membrane potential (MMP) collapse, reduced ATP generation, and disrupted calcium homeostasis, which collectively impair intracellular signaling and accelerate lymphocyte functional decline [69,70]. In aging CD8^+^ T cells, ROS-induced mtDNA leakage stimulates the cGAS–STING–interferon regulatory factor 3 (IRF3) axis, amplifying systemic inflammation, a pathological decline that can be effectively mitigated by nicotinamide mononucleotide supplementation [71]. This accumulation of damaged organelles is further driven by defective cell-autonomous autophagy in aged CD4^+^ T cells [65]. A parallel breakdown in mitophagy is observed in aging macrophages. Under physiological conditions, guanylate-binding protein 1 (GBP1) promotes autophagy to clear damaged mitochondria; however, in metabolic stress models, macrophages exhibit a downregulation of GBP1 that leads to defective mitophagy and increased SASP secretion via the AMP-activated protein kinase (AMPK)-p53 pathway [72]. This metabolic and mitochondrial reprogramming is further driven by mitogen-activated protein kinase (MAPK)-NF-κB, mTOR-hypoxia inducible factor-1α (HIF-1α)-glycolysis, and Toll-like receptor 4 (TLR4)-STAT1 pathways [73,74], which collectively transform senescent macrophages into pro-inflammatory M1 phenotypes characterized by suppressed mitochondrial respiration and elevated glycolysis, an inflammatory state induced during aging by matricellular proteins such as SPARC [70]. This chronic macrophage activation can be counteracted by metabolic modulators like citrulline, which restrain inflammatory pathways to counter immunosenescence [74]. Pathological conditions heavily aggravate this baseline mitochondrial damage. In Alzheimer’s disease models, amyloid-beta (Aβ) suppresses the microglial SIRT1/NFE2L2 pathway, resulting in elevated ROS, MMP collapse, and impaired phagocytosis, a deficit that can be therapeutically alleviated by quercetin supplementation [75]. Conversely, microglial activation can be compromised by the adverse effects of carboxylic acid non-steroidal anti-inflammatory drugs, which induce metabolic alterations that worsen mitochondrial dysfunction and oxidative stress [76]. In the context of neuro-HIV, the viral transactivator of transcription (TAT) protein upregulates miR-505, which directly inhibits the mitochondrial deacetylase SIRT3, thereby weakening enzymatic antioxidant defenses [77]. Across aging tissues, excessive ROS production is tied to a multidimensional collapse of MQC systems, characterized by the decreased expression of mitochondrial 8-oxoguanine DNA glycosylase (mtOGG1), impaired ATP synthesis, and the inhibition of the mammalian target of rapamycin (mTOR)/transcription factor EB and PINK1/Parkin pathways [63]. Although mtOGG1 downregulation occurs in both aged male and female mice, therapeutic restoration of its activity reduces neuroinflammation by regulating cytosolic mtDNA exclusively in males, highlighting poorly understood sex-specific regulatory mechanisms [78]. Mechanistically, ROS also activate c-Jun N-terminal kinase (JNK) via retrograde mitochondria-to-nucleus signaling. JNK activation prevents the physiological suppression of p53-binding protein 1 (53BP1) during double-strand break end resection, leading to the shedding of cytoplasmic chromatin fragments (CCFs) that stimulate NF-κB and cGAS–STING signaling to drive SASP expression [79]. In the central nervous system, leaked mtDNA fuels the cGAS/STING pathway across microglia, oligodendrocytes, and astrocytes, driving sustained type I interferon production and chronic neuroinflammation, a mechanism highly relevant in brain injuries [80]. This neuroinflammatory environment compromises vascular barriers; microglial-derived tumor necrosis factor-alpha (TNF-α) upregulates vascular cell adhesion molecule 1 and intercellular adhesion molecule 1 in the aged subventricular zone, facilitating peripheral T-cell infiltration [81]. Once recruited by microglial C-C motif chemokine ligand 3 (CCL3), these invading CD8^+^ memory T cells secrete CCL4, CCL5, IFN-γ, FasL, and X-C motif chemokine ligand 1, aggravating localized neuroinflammation [81]. While clinical data confirm that chronic systemic inflammation in older adults significantly mediates psychomotor slowing, the exact molecular links mapping these inflammatory events to neurological dysfunction remain incomplete [82]. Ultimately, SIRT1 functions as a key metabolic gatekeeper by negatively regulating T cell activation and suppressing glycolysis through the deacetylation of phosphoglycerate mutase-1 [65]. The combined impact of impaired MQC, metabolic skewing, and persistent DAMP leakage establishes a self-reinforcing pathogenic cycle that drives immune decline, rendering therapeutic strategies aimed at preserving mitochondrial homeostasis and restoring MQC highly promising interventions to improve immune competence in older people.

## 3. Altered Intercellular Communication

Bidirectional communication between the endocrine and immune systems occurs through neuroendocrine regulation via the hypothalamus–pituitary–thyroid (HPT) axis and the exchange of soluble signaling molecules, such as cytokines, chemokines, and thyroid hormones, between immune cells. This intercellular communication allows immune cells to influence systemic thyroid hormone homeostasis, while, in turn, thyroid hormones regulate innate and adaptive immune cell functions [83]. In the context of immunosenescence, neuroendocrine control plays a relevant role in shaping age-related changes in immune function. Age-related dysfunction of central organs, particularly the thymus and pineal gland, is associated with alterations in immune–endocrine interactions. Notably, melatonin and thymic hormones such as thymulin not only inhibit age-related changes in immune and neuroendocrine function but also modulate the proliferative and differential potential of stem cells, thereby influencing the development and clinical outcomes of age-related diseases [84]. Similarly, in aged rats, high levels of corticotropin-releasing hormone decrease splenic NK cell cytotoxicity, contributing to weakening the immune system. An age-related decrease in pineal melatonin levels also promotes thymic involution and a reduction in lymphocyte numbers in the spleen and peripheral blood. Conversely, pineal gland transplantation or melatonin supplementation enhances immune function in mice, highlighting the potential role of neuroendocrine interventions in mitigating immune aging [85].

Immune dysregulation may also arise from age-associated alterations in neuroendocrine–immune interactions, as reflected by changes in hormonal rhythms and immune cell profiles [86]. For instance, melatonin reduces choroidal neovascularization (CNV) in aged mice by blocking the IL-10/STAT3-mediated polarization of microglia and macrophages toward a proangiogenic M2-like phenotype, providing a useful target to treat CNV and other age-related diseases [85]. Helper/inducer T cells exhibit positive relationships with daytime cortisol levels in older subjects, unlike total B cells that show an inverse correlation. On the other hand, daytime cortisol levels correlate positively with suppressor/cytotoxic T cells and negatively with total T lymphocyte numbers [86]. These age-dependent alterations in the circadian relationships between cortisol and immune cell populations differ from those observed in younger individuals, although their functional implications for immune homeostasis and immunosenescence remain to be fully elucidated.

## 4. Chronic Inflammation

The state of persistent, low-grade, sterile inflammation that characterizes aging and accompanies immunosenescence is marked by an increase in several circulating inflammatory mediators [87], underpinning the progressive establishment of chronic inflammation. Among these, IL-6 is one of the most relevant biomarkers, while TNF-α has recently been indicated as a stronger indicator of chronic inflammation [88].

As people age, high levels of TNF-α, IL-6, and interleukin-1 receptor antagonist are associated with higher concentrations of circulating mtDNA, highlighting the contribution of mitochondrial damage to systemic and peripheral inflammation [89]. In the auditory system, age-associated inflammation of the cochlea and cochlear nucleus appears to be mediated primarily by the nuclear factor kappa B (NF-κB) pathway and the NOD-like receptor pyrin domain-containing protein 3 inflammasome. This inflammatory response is driven by macrophages, microglia, and complement component 1q deposition and contributes to the development of age-related hearing loss [90]. In addition to the nervous system, aging is also associated with profound inflammatory remodeling of visceral adipose tissue (VAT). Aged VAT shows an accumulation of tissue-resident memory γδ T cells, characterized by a CD44^hi^CD62L^low^CD69^+^ phenotype, and a higher proportion of pro-inflammatory IL-17A^+^-producing cells. These γδ T cells promote chronic inflammation by releasing IL-17A, which stimulates IL-6 production by preadipocytes and adipose-derived stem cells [91].

Targeting sterile chronic inflammation helps mitigate or partially reverse immunosenescence. Several lifestyle and pharmacological interventions have shown promise in this regard. For instance, coffee consumption reduces senescent T cells and suppresses SASP by inhibiting the Janus kinase/signal transducer and activator of transcription and mitogen-activated protein kinase (MAPK) signaling pathways, suggesting a possible anti-immunosenescence effect [92]. Additionally, administration of the senolytic combination of dasatinib with quercetin decreased circulating SASP factors in diabetic patients, although the exact mechanism is yet unknown [93]. Zinc supplementation has also been shown to reduce IL-6, Th1/Th17-associated inflammatory cytokines, and monocyte chemoattractant protein 1, while increasing the proportion of naïve CD4^+^ T cells [94]. Dietary interventions also modulate chronic inflammation through the gut–immune axis. Supplementation with *Lactobacillus paracasei* KW3110 has been reported to modify gut microbial composition and to lower circulating pro-inflammatory cytokines, as well as the frequency of IFN-γ-producing CD4^+^ T cells in the lamina propria of the small intestine, attenuating intestinal and ocular inflammation [95]. Likewise, high-fiber diets increase butyrate-producing bacteria in the gut, which can inhibit microglial pro-inflammatory signals and lower chronic neuroinflammation. Finally, physical exercise reduces the levels inflammatory mediators and blunts systemic inflammation [96]. However, the translational relevance of these dietary interventions to humans remains uncertain, and further studies are needed to determine whether specific dietary patterns can effectively modulate chronic inflammation and immunosenescence in older individuals.

## 5. Effects of Aging on Systemic Immunity in Cancer

### 5.1. Aging and Systemic Immunity in Cancer

**Figure 3:**
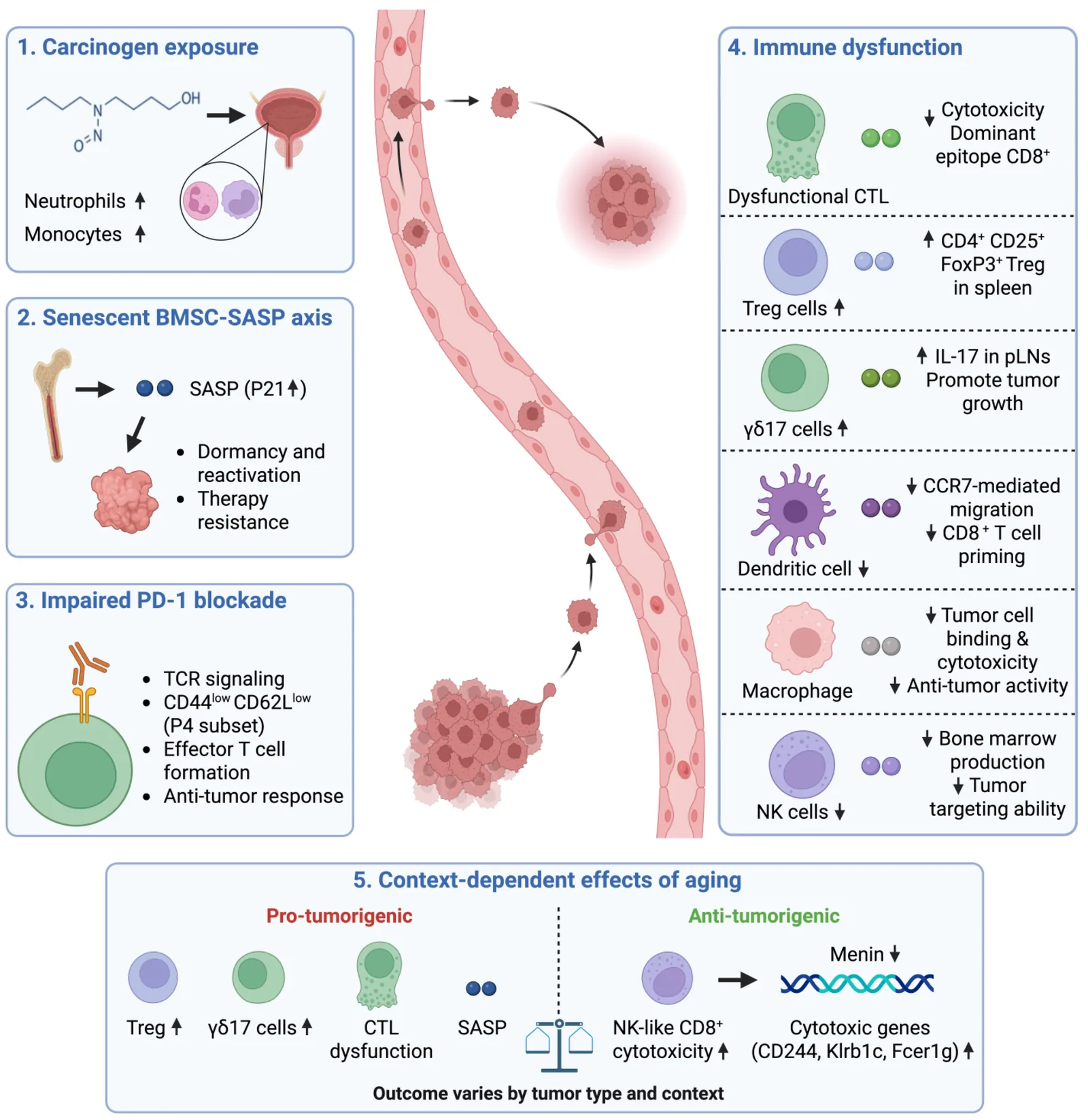
Age-associated mechanisms promoting tumor progression and influencing antitumor immunity. Aging enhances cancer development through increased carcinogen exposure, the development of a senescent bone marrow mesenchymal stem cell and senescence-associated secretory phenotype axis, impaired responses to PD-1 blockade, and immune dysfunction affecting cytotoxic T cells, regulatory T cells, γδ17 cells, DCs, macrophages, and NK cells. Depending on the tumor type and microenvironment, aging exerts both pro-tumorigenic and anti-tumorigenic effects, ultimately shaping tumor progression and therapeutic responses. *Abbreviations*: BMSC, bone marrow stromal cell; CCR7, C-C motif chemokine receptor 7; CD 25^+^, cluster of differentiation 25 positive; CD244, cluster of differentiation 244; CD4^+^, cluster of differentiation 4 positive; CD44, cluster of differentiation 44; CD62L, cluster of differentiation 62 ligand; CD8^+^, cluster of differentiation 8-positive; CTL, cytotoxic T lymphocyte; Fcer1g, Fc epsilon receptor Ig; FoxP3^+^, forkhead box P3; IL-17, interleukin-17; Klrb1c, killer cell lectin-like receptor subfamily B member 1C; NK, natural killer; p21, cyclin-dependent kinase inhibitor 1A; PD-1, programmed cell death protein 1; TCR, T-cell receptor; pLNs, peripheral lymph nodes; SASP, senescence-associated secretory phenotype; Treg, regulatory T cell; γδ T cells, gamma-delta T cells. Created with BioRender.com accessed on 5 September 2026.

The incidence of cancer increases markedly with age, likely reflecting the progressive accumulation of genetic, metabolic, and immunological alterations that increase vulnerability to tumors. Figure 3 summarizes the major effects of aging on systemic immunity in tumor patients. Aging can influence both tumor development and the immune landscape of established tumors. For instance, exposure to the bladder cancer carcinogen N-butyl-N-(4-hydroxy-butyl) nitrosamine induces bladder carcinogenesis with increased neutrophil and monocyte infiltration as people age, although the chemical mechanism is not yet known [97]. Tumor resistance to therapy can also be enhanced by aging through alterations in the cellular components of the tumor microenvironment and systemic immune response. Senescent bone marrow mesenchymal stem cells promote the dormancy of breast cancer cells that have metastasized to bone and reduce their proliferative capacity through the SASP-mediated induction of p21, thereby facilitating tumor cell persistence and potentially contributing to treatment resistance [98]. The tumor potentially develops resistance through the age-related decrease in effector T cells, which are the target for several therapies. In aged mice, programmed cell death protein 1 (PD-1) blockade therapy is less effective because naïve CD8^+^ T cells display higher levels of CD45RB protein, which weakens T-cell receptor (TCR) signaling and decreases the formation of the CD44^low^CD62L^low^ (P4) T-cell subset. This subset derives from naïve T cells, shows strong expression of genes involved in one-carbon metabolism, and subsequently differentiates into tumor-reactive effector T cells in vivo. Interestingly, potent immune activation by non-self-antigens can restore CD8^+^ T cell antitumor activity by reversing this TCR inhibition [99]. Despite these preclinical findings, clinical trials indicate comparable effectiveness of anti-programmed cell death protein 1/anti-programmed cell death protein ligand 1 (anti-PD-1/anti-PD-L1) therapies across different age groups. However, older patients experience a higher incidence of immune-related adverse events, increasing the likelihood of treatment discontinuation [100].

Clinical evidence indicates that the preserved efficacy of immune checkpoint inhibitors (ICIs) in older patients does not necessarily indicate an unchanged immune response. A meta-analysis of 30 randomized phase II/III trials including 17,476 patients found a similar overall survival benefit from ICIs in patients aged ≥65 years and those aged <65 years, with no significant interaction between age and treatment efficacy [101]. In contrast, analysis of 47,513 patients reporting immune-related adverse events in the FDA Adverse Event Reporting System showed a higher risk of reported immune-related adverse events in patients aged 65–74 years and 75–84 years than in patients aged 18–64 years, although the pattern differed among individual organ systems [102].

Age-related changes in immune cell populations other than T cells contribute to these differences in treatment response and toxicity. Aging is associated with changes in B-cell composition and function, chronic inflammatory signaling, and the expansion of age-associated B-cell populations [9]. B cells also contribute to immune-related toxicity during checkpoint blockade, as alterations in the B-cell compartment have been associated with the development of immune-related adverse events. In particular, impaired regulatory B-cell responses increase an autoreactive T-cell activity and autoantibody production following PD-1/PD-L1 blockade. Dendritic cells also influence the response to checkpoint inhibition because PD-L1 expressed by dendritic cells regulates T-cell priming, and dendritic cell signatures have been associated with improved survival in patients receiving PD-L1 blockade [8]. Therefore, age-related changes in B cells, dendritic cells, macrophages, and other myeloid populations, together with altered inflammatory and cytokine signals, influence both the antitumor activity and toxicity of ICIs in older patients.

This phenomenon is explained by the fact that several immune cell types, including B cells, macrophages, T cells, and DCs, express PD-1. Consequently, the effects of PD-1 blockade on non-T cells populations should also be considered when evaluating its therapeutic efficacy and toxicity in older patients [103]. Interestingly, the relationship between aging and cancer outcomes is highly context-dependent and varies substantially among tumor types. Although aging is typically considered a risk factor for cancer mortality, reduced immune checkpoint activity within the TME is linked to poorer outcomes in many cancer types, including breast cancer and acute myeloid leukemia, likely because impaired checkpoint signaling compromises adaptive immune responses. This age-related molecular pattern and its prognostic impact have not been observed in glioblastoma or colorectal adenocarcinoma [104].

### 5.2. Age-Associated Gene Signatures as Prognostic and Predictive Tools

Prognostic models based on aging-related genes (ARGs) have been developed in many cancers, such as colorectal cancer, melanoma, lung cancer, glioblastoma, renal cell carcinoma, acute myeloid leukemia (AML), ovarian cancer, hepatocellular carcinoma (HCC), and head and neck squamous cell carcinoma (HNSCC) [105,106,107,108,109,110].

Despite differences in the ARGs, these models generally associate poorer responses to ICIs with high-risk molecular profiles. Similarly, predictive models based on cell senescence-related long non-coding RNAs have been developed for HNSCC [111], glioma [112], and colorectal cancer [113]. Furthermore, a recent study proposed a model based on aging cancer-associated fibroblast (CAF)-related genes to predict prognosis and response to immune checkpoint blockade in low-grade gliomas [114]. Finally, the combined CellAge and TME scores, based on the expression of cellular aging- and TME-associated genes, have been used as independent predictors of survival and immunotherapy response in cancer. Nevertheless, further research is needed to determine whether these models provide consistent and clinically meaningful information within the same type of tumor and across independent patient cohorts [115].

Integrating transcriptomic, proteomic, epigenomic, and metabolomic data may provide a more comprehensive view of how age-related processes are regulated at different molecular levels. Such an approach helps identify age-associated changes in gene expression together with corresponding alterations in protein abundance, epigenetic regulation, and cellular metabolism, thereby providing a clearer understanding of their contribution to tumor development and immune dysfunction. Multi-omics analysis also helps identify molecular signatures that distinguish normal aging from pathological changes associated with cancer and could reveal potential biomarkers and/or therapeutic targets in older patients [116].

### 5.3. Age-Associated Dysfunction of Specific Immune Cell Populations in Cancer

While the exact link between aging and tumor progression is not completely deciphered, some evidence indicates that aging promotes tumor development by weakening the immune system, manifesting via the expansion of immunosuppressive regulatory T cells (Tregs), the accumulation of protumor γδ17 T cells, and the impaired function of cytotoxic T lymphocytes (CTLs) (Figure 3) [3,4]. Early research investigated whether the age-associated drop in CTL cytotoxicity was caused primarily by intrinsic cellular defects or by decreased cell numbers. A recent study indicated that both mechanisms contribute to CTL cell dysfunction during aging [117]. In aged mice, the relative frequency of dominant and subdominant epitope-recognizing CD8^+^ T cells increases despite an overall reduction in CD8^+^ T cell numbers. However, dominant epitope-recognizing CD8^+^ T cells show decreased cytotoxicity compared with those from young mice, whereas subdominant epitope-recognizing cells retain their functional capacity, increasing tumor growth during aging [118]. Mitochondrial dysfunction further compromises antitumor T-cell activity. Aging activates mitochondrial sphingosine kinase 2/sphingosine-1-phosphate signaling in T cells, leading to histone deacetylase 1/2 suppression and CerS6-mediated C14 ceramide accumulation [119]. These alterations impair mitochondrial function and antitumor effectiveness in activated CD8^+^ T lymphocytes by suppressing protein kinase A activity and decreasing inhibitory S637 phosphorylation of dynamin-related protein 1 (Drp1), thereby promoting Drp1-driven mitophagy. High levels of the microtubule-associated protein 1 light chain 3 (LC3) further recruit autophagosomes to enhance mitophagy, although the precise molecular mechanism is unclear [119]. Aging also enhances IL-17 production within the T-cell areas of peripheral lymph nodes, promoting the development of γδ17 cells, a pro-inflammatory subtype of γδT cells. These cells become activated in tumor-draining lymph nodes, subsequently migrate into tumors, and favor tumor growth (Figure 3). However, despite their altered abundance and function, the gene expression patterns and normal functions of aged γδT cells remain largely preserved [120]. Similarly, aged lung cancer models show an increased frequency of splenic CD4^+^CD25^+^Foxp3^+^ Tregs, which is associated with both aging and tumor development, although the mechanisms behind this expansion remain unclear.

Along with T cells, other immune cells also have a role in the aging-related acceleration of tumor growth. Aged DCs show poor CD8^+^ T cell priming in vitro, decreased migration to draining lymph nodes, and lowered activation of T cell immune responses in vivo, partly because of altered CCR7-dependent chemotactic signaling [121]. Additionally, autologous DC vaccinations combined with anti-CD134 monoclonal antibodies can restore antitumor responses, suggesting that co-stimulatory deficiencies contribute to age-related DC dysfunction [122]. Macrophage function is similarly compromised during aging, with decreased production of inducible nitric oxide synthase, TNF, IL-1, nitric oxide, and oncostatin-M as well as reduced tumor cell binding and cytotoxicity [122]. These changes collectively weaken macrophage-mediated antitumor activity. While cancer incidence increases with age, tumor growth and metastatic spread often proceed at a slower rate in aged hosts. This paradox has been attributed to several age-related changes within the TME, including reduced tumor cell proliferation, enhanced apoptotic cell death, and diminished angiogenesis. Additionally, age-associated alterations in immune function, such as impaired NK-cell activity due to decreased bone marrow production, contribute to this differential tumor behavior, though the net effect on tumor progression remains complex and context-dependent [123]. The immune system also seems to play a role in the aging-related slowdown of tumor growth. When activated in vitro, CD8^+^ T cells from older mice show increased antigen-independent cytotoxicity and acquire features resembling NK cells [124]. This change results from the age-related downregulation of the protein menin, which increases the expression of cytotoxic genes like CD244, Fc fragment of IgE receptor Ig, NK-associated molecules, and killer cell lectin-like receptor B1c (Figure 3). This enhanced cytotoxic program contributes to inhibited tumor metastasis in aged mice. Therefore, aging has two distinct, context-dependent impacts on tumor biology through different cellular, metabolic, and immunological pathways.

### 5.4. Therapeutic Strategies: Preclinical and Translational Approaches

The distinct immune landscapes observed in young and old cancer patients provide an opportunity to develop age-adapted therapies. Although there are still many unanswered questions about the interaction among aging, tumor progression, and systemic immunity, preclinical evidence indicates that some therapeutic approaches are particularly effective in older people. For instance, the anti-angiogenic agent TNP-470, apoptosis-inducing drugs, hydrocorotisone, adriamycin, and immunomodulators like Bacillus Calmette-Guérin have shown improved effectiveness in older mice compared with younger ones [125]. Advances in therapeutic engineering further enable the development of personalized anticancer approaches tailored to the immune characteristics of older patients. Modified fusokines represent one such strategy. In aged mice models of glioblastoma, subcutaneous vaccination with GL261 and CT2A glioblastoma cells engineered to express GIFT-7, a fusion protein comprising IL-7 and granulocyte-macrophage colony-stimulating factor, significantly reduced tumor size and increased survival. This effect was achieved by activating DCs, which helped recruit systemic T cells to the brain tumor site. Remarkably, treated mice developed a CD4^+^ Th17-dominant immune response that protected against glioma recurrence [126]. Rather than uniformly promoting tumor progression, immunosenescence can either impair antitumor surveillance and therapeutic efficacy or, under specific circumstances, generate immune responses that restrict tumor growth and metastasis. These age-specific mechanisms represent a promising approach for developing personalized treatments based on the biological characteristics of older people, although their clinical safety, efficacy, and translational applicability still need to be confirmed.

## 6. Age-Associated Remodeling of the TME

Aging of the TME is characterized by the accumulation of pro-inflammatory SASP factors, increased recruitment of immunosuppressive cells, metabolic abnormalities, and extracellular matrix remodeling. These features constitute a localized manifestation of systemic immunosenescence rather than an isolated event. In particular, the SASP promotes a pro-inflammatory and immunosuppressive TME, contributing to the chronic inflammation that characterizes systemic immunosenescence. In aged mice with intraocular melanoma, CD163^+^ tumor-associated macrophages (TAMs) can be divided into two functional groups: one promotes immunosuppression by expressing lymphocyte antigen 6 complex, locus C, while the other supports tumor growth by expressing peroxisome proliferator-activated receptor gamma [127]. Moreover, age-associated TAMs promote tumor progression and metastasis. Different SASP factors, such as CCL2, CCL8, bone morphogenetic protein-2, and vascular endothelial growth factor A, are secreted by p16INK4a-expressing macrophages in aging mice [126,128]. SASP cytokines within the aging TME are not exclusively produced by immune cells but can also be secreted by cancer and stromal cells. Similarly, tumors use CXCL2 to induce the aging of circulating neutrophils and their migration into the TME to promote tumor progression [129]. Cancer-associated fibroblasts (CAFs) represent another major source of inflammatory and tumor-promoting factors in the aging TME. Among the mediators released by CAFs are ROS, L-lactic acid, secreted frizzled-related protein (SFRP) 1, growth differentiation factor 15 (GDF15), CCL2, and IL-8 [130,131,132,133]. GDF15 released by senescent CAFs promotes tumor invasion and proliferation in colorectal cancer through the activation of the phosphoinositide 3-kinase and mitogen-activated protein kinase (MAPK) pathway [134]. Similarly, CAFs-derived IL-6 increases the aggressiveness of pancreatic and cervical cancers. Thus, age-associated alterations in the TME arise from the coordinated contribution of immune and stromal cells, which both shape and respond to the inflammatory, metabolic, and immunosuppressive environment surrounding the tumor.

### 6.1. Cell Types Involved in Age-Related Remodeling of the TME

#### 6.1.1. T Cells

As described earlier, T cell senescence is frequently followed by a progressive reduction in its functionality. Within the TME, this dysfunction leads to immunosuppression through the downregulation of effector and costimulatory molecules, the induction of premature senescence in responder T cells and adaptive Treg cells, and the inhibition of effector T cells and DC activity. Although the exact molecular mechanisms are not fully understood, several pathways have been proposed, including the ataxia-telangiectasia mutated (ATM)-mediated DNA damage response, activation of the MAPK and STAT1/3 pathways due to glucose competition caused by the high glucose uptake of Treg cells, and the accumulation of tumor-derived metabolites, such as adenosine, cyclic AMP, lactate, and cytokines [135]. Both tumor cells and Treg cells stimulate T-cell senescence through ATM and MAPK signaling, and simultaneous inhibition of these pathways has been shown to improve treatment responses to immunotherapy in vivo [136]. However, inhibiting the MAPK pathway in senescent T cells carries the risk of disrupting the physiological functions of healthy T cells that do not require such blockade [136]. In this context, TLR8 activation not only directly inhibits the MAPK pathway but also suppresses Treg glucose metabolism through mTOR inhibition, reducing immunosuppression and T cell senescence without altering normal T cell function. Thus, TLR8 could be a more effective therapeutic target than MAPK pathway inhibitors. Lipid metabolism also contributes to the senescence of responder T cells through several interconnected mechanisms, including fatty acid droplet formation, activation of the STAT1/3 and MAPK pathways, increased cytosolic phospholipase A2 expression, and Treg involvement [137].

Aging of the TME can also directly drive CD8^+^ T cell dysfunction. In aged mice, the TME promotes the differentiation of CD8^+^ T cells into a dysfunctional population termed tumor-infiltrating age-associated dysfunctional cells. These cells express high levels of PD-1, thymocyte selection-associated high mobility group box, and IL-7R, but have very low cytotoxic activity. This dysfunction is induced by fewer NK cells in the aged TME, which hinder the recruitment of conventional type 1 DCs through antigen presentation and XCL1 signaling, reducing the activation of CD8^+^ T cells. Of note, CD40 agonists can increase myeloid cell numbers and activity, which restores cDC1 function and enhances CD8^+^ T cell cytotoxicity, providing a potential therapeutic option [138].

Recent single-cell multi-omic approaches further highlight the importance of mitochondrial heterogeneity in immune cell states. A mitochondrial single-cell ATAC-seq (mtscATAC-seq) method has been described that enables simultaneous profiling of mtDNA and chromatin accessibility in individual cells. This approach links mitochondrial genetic variation with cell state and chromatin differences and identifies immune cell subpopulations with distinct mitochondrial vulnerabilities [139]. Such approaches could be particularly useful for resolving heterogeneous CD8^+^ T-cell states during aging and determining whether mitochondrial alterations are associated with impaired antitumor function. These observations have also prompted the development of strategies aimed at selectively targeting Tregs. Suppression of vascular endothelial growth factor receptor 2 in tumors increases the release of exosomal miR-3200-3p, which targets damage-specific DNA binding protein 1 in Tregs and induces their senescence, providing a new Treg-targeted treatment [140]. Similarly, the immunoglobulin-like transcript 4 blocker PIR-B has been proposed as a strategy to target tumor-associated lipid metabolism. PIR-B reverses T-cell senescence and increases T cell antitumor responses by disrupting MAPK–ERK1/2-mediated lipid accumulation in tumors [141]. Finally, extracellular ATP, another extracellular metabolite abundant in the TME, triggers stress-induced premature senescence in responder T cells via the purinergic receptor P2X7 ion channel. However, eATP exerts the opposite effect on intratumoral myeloid subsets and on the tumor, indicating that T cell-specific P2X7 inhibitors are required to selectively restore T-cell function [142].

#### 6.1.2. B Lymphocytes

Evidence on the role of B cells in the TME is quite limited. Most studies on peripheral blood B lymphocytes have investigated the effects of senescence on tumor-bearing hosts in humans and animals. With advancing age, B1 cells (CD5^+^ CD19^+^) can transition into 4-1BBL^+^ B cells (4BL cells), an innate-like, immature B cell population that declines over time. These cells can enhance tumor cell killing upon low-threshold stimulation by activating granzyme B (GrB^+^)-expressing CD8^+^ T lymphocytes through the mTNFα-TNFR2/4-1BBL axis [143]. Another group of B cells, characterized by B7-DC (PD-L2/CD273) expression, markedly enhances the induction of Th1 and Th17 responses [144]. Additionally, B cells within the TME have been implicated in cancer metastasis [145]. Notably, TME and in vivo testing are still required to validate these findings.

#### 6.1.3. Neutrophils

Tumor-associated neutrophils (TANs) in the TME are important regulators of tumor progression and can exert either protumor or antitumor effects via the release of type I IFN and ROS. Their antitumor functions engage neutrophil extracellular trap (NETs) formation, degranulation, and phagocytosis. TANs are commonly classified into N1, generally associated with pro-inflammatory and antitumor activity, and N2, related to anti-inflammatory and tumor-promoting functions [5]. Aging alters several functional properties of TANs. Compared with younger people, older cancer patients show higher polymorphonuclear leukocyte phagocytosis but lower intracellular killing power, which limits antitumor immunity and therapeutic treatment responses [146]. Furthermore, tumor-derived nicotinamide phosphoribosyl transferase allows the accumulation of aging-associated neutrophils (Naged, CXCR4^hi^ CD62L^low^). These cells are distinct from conventional N1 or N2 subsets and release metastasis-promoting NETs enriched in mtDNA. In mice, breast cancer lung metastasis is decreased by blocking this pathway [147]. Targeting aged neutrophils is therefore emerging as a potential therapeutic strategy. CXCR2 inhibitors are effective in treating breast cancer in clinical contexts [148]. Finally, in preclinical models, anti-B-cell lymphoma-extra-large drugs in combination with granulocyte-macrophage colony-stimulating factor have been shown to selectively eradicate aged TANs while preserving antitumor ANs and normal neutrophils [149]. However, platelet toxicity associated with treatment still represents a limitation.

#### 6.1.4. Natural Killer

Natural killer (NK) cells contribute to antitumor immunity primarily via direct cytolysis, chemokine secretion, and cytokine production [5]. Despite their established role in tumor surveillance, limited research has been devoted to investigating the relationship between NK cell senescence and tumor. Most available evidence has focused on leukemia, rather than the TME or solid tissues [150]. Thus, the exact role of NK cell senescence in the aging TME and its impact on tumor progression is yet unknown.

#### 6.1.5. Macrophages

Macrophages within the TME, collectively referred to as TAMs, are highly plastic cells that can acquire distinct functional states. Additionally, they can be categorized into M1 (anti-tumorigenic) and the M2 (pro-tumorigenic) subtypes [5]. Aging induces macrophage differentiation more towards M2 type through mechanisms involving IL-8 and Bcl3 signaling within the TME. Consistent with this, higher M2-type macrophages have been observed in older adults with breast cancer [151]. Additionally, aged TMEs are characterized by more dysfunctional or exhausted macrophages. However, although their numbers increase, these cells fail to effectively improve cytotoxic T-cell responses or antitumor ability [152]. Furthermore, senescent TAMs show reduced anticancer efficacy because of decreased production of nitric oxide, TNF, IL-1, and oncostatin M, and poor cytotoxicity and impaired tumor-cell binding [153]. Of note, the effectiveness of IL-2/anti-CD40 immunotherapy is negatively impacted by the accumulation of CD11b^+^F4/80^+^Ly6G^−^Siglec-F^−^ macrophages in aged mesothelioma TMEs. Macrophage depletion has been shown to improve treatment responses in older mice, although the underlying processes need to be clarified [154]. Lifestyle interventions also influence age-associated macrophage dysfunction. For instance, exercise has been proposed to ameliorate age-related alterations in macrophage polarization and function, but the exact mechanisms by which this occurs have not yet been fully clarified.

## 7. Effect of Aging on Cancer Immunotherapy

The incomplete understanding of the relationship between immunesenescence and the aging of the TME has hampered the development of efficient immunotherapies [155]. Although immunosenescence and the age-related remodeling of the TME are expected to reduce treatment efficacy in older patients, accumulating evidence indicates that several immunotherapies retain substantial clinical activity across age groups. For instance, PD-1 inhibitors, such as pembrolizumab and cemiplimab, have shown favorable safety and efficacy profiles in older and immunocompromised people with metastatic cutaneous squamous cell carcinoma (cSCC) and non-small cell lung cancer (NSCLC) [156]. Similarly, CAR-T cell treatment has shown low toxicity and efficacy in older patients with multiple myeloma. The type, frequency, and severity of immune-related adverse events (irAEs) from cytotoxic T lymphocyte-associated antigen-4 (CTLA-4) or PD-L1 inhibitors are also broadly similar across age groups, with patients over 75 experiencing benefits comparable to those observed in younger individuals without excess toxicity [157]. Similar results have been reported for other immunotherapy strategies. For example, age does not appear to affect the effectiveness of intravesical Bacillus Calmette-Guérin therapy in patients with bladder cancer [158]. These observations highlight that chronological age alone should not be considered a contraindication to immunotherapy, but further investigation is warranted.

Importantly, immune aging does not necessarily result in a uniform decline in antitumor immunity or consistently promote tumor progression. Although aging impairs NK-cell activity, T-cell function, and immune surveillance, some cytotoxic immune responses remain preserved or enhanced in certain older individuals. The effects of immune aging are therefore likely to depend on the cellular composition and functional state of the TME, as well as on the specific type of cancer. This heterogeneity explains why older patients can show comparable responses to some immunotherapies, whereas reduced efficacy or increased toxicity is observed in other tumor types [159].

However, age-related variations between older and younger patients become apparent when irAEs are categorized by affected organ systems. Multiple irAEs and grade ≥ II events are more frequent in older people receiving anti-PD-L1 treatment for solid tumors [160]. Additionally, the toxicity profile of combination immunotherapy differs according to age. Older adults treated with nivolumab (monotherapy or in combination with ipilimumab) for metastatic renal cell cancer show antitumor responses comparable to those of younger patients, although they have a greater prevalence of gastrointestinal irAEs [161]. Similarly, irAEs are more frequent among patients over 70 receiving camrelizumab in addition to chemotherapy [162]. To reduce this side effect, blocking the IL-6 receptor together with PD-L1 inhibition has been shown to decrease irAEs in solid tumors. However, there are still not enough clinical data to determine its safety and effectiveness in older patients.

Treatment optimization also requires personalized dosing that accounts for patient-specific pharmacokinetic profiles, balancing the response and adverse effects of immunotherapy. Pharmacokinetically guided pembrolizumab administration has shown encouraging results with manageable toxicity [163]. However, studies specifically designed for different age groups have not yet been conducted. Since the TME in older patients is highly complex, this approach is still worth investigating. Interestingly, although treatment effectiveness does not appear to differ according to age, genitourinary cancer patients older than 75 years who develop irAEs tend to have better survival outcomes [164]. This association has not been reported in most other cancer types and related to the unique features of genitourinary tumors. Conversely, a recent study found that patients over 70 with solid tumors receiving anti-PD-L1 therapy had a lower incidence of all-grade irAE [165]. These contrasting results require validation in larger populations.

While some studies report comparable results across age groups, others indicate lower treatment efficacy in specific tumor types. For instance, Bacillus Calmette-Guérin or IFN-α treatment appears to be less effective in older patients (>80 years) with bladder cancer [166]. Similarly, in glioblastoma, a large number of PD-1-negative CD8^+^ CD28-T cells with cytotoxic dysfunction make anti-PD-1 treatment less effective [167]. Aged mice show reduced NK cell development, cytotoxicity, and infiltration. This impairs the recruitment of DCs to tumor-draining lymph nodes, which in turn reduces the activation of tumor antigen-specific CD8^+^ T lymphocytes and compromises the efficacy of anti-PD-L1 treatment. Instead, infusion of mature NK cells into aged mice has been shown to improve T cell infiltration into the TME after immunotherapy and to restore treatment responsiveness [168]. Age-related immune dysfunction can also impair other immunotherapeutic approaches, including anti-PD-1/PD-L1, anti-CD4 mAb, anti-OX40 antibody, endotoxin injection, and CTLA-4 blockade. These effects are linked with T-cell exhaustion and dysfunction within the aged TME [169]. Moreover, increased Tregs in aged tumors reduce CTL activity [169]. The combination of a CpG oligonucleotide adjuvant with Treg depletion has been shown to improve the antitumor immune response, although this approach does not produce long-term immune memory in aged mice [170]. Metabolic interventions also improve immunotherapy responses. L-arginine supplementation promotes CTL infiltration, which potentiates the efficacy of PD-1 antibody in older CT26 colon cancer models. This advantage is tumor-specific and is not observed in MC38 models, indicating tumor heterogeneity [171].

The effects of aging on antitumor immunity are not uniform across tumor types, because the composition and function of the TME differ substantially between cancer types. A common feature across several tumors is the accumulation of inflammatory and immunosuppressive changes, including altered antigen presentation, impaired effector T-cell function, the expansion of suppressive myeloid populations, and the remodeling of stromal cells. However, the relative contribution of these mechanisms appears to be cancer-specific. In triple-negative breast cancer (TNBC), aging is associated with reduced IFN-α/IFN-γ signaling and impaired antigen presentation, resulting in a less inflamed TME and reduced response to PD-L1 or CTLA-4 blockade in aged mouse models; similar reductions in IFN signaling are observed in tumors from older patients [172]. In contrast, studies of melanoma have highlighted a more prominent contribution of the aged stromal compartment to tumor progression and therapeutic resistance. Adipocyte-derived lipids can be transferred to melanoma cells through fatty acid transport proteins, promoting tumor growth and invasion, while lipid metabolic reprogramming has also been implicated in resistance to BRAF-targeted therapy [173]. Age-related changes in stromal composition and extracellular matrix organization may further influence tumor progression by modifying the physical properties of the tissue microenvironment. Collagen fiber remodeling can facilitate tumor cell invasion, highlighting also the contribution of extracellular matrix architecture to malignant dissemination [174]. In prostate cancer, aging is accompanied by increased inflammatory and chemokine-related gene expression, changes in extracellular matrix organization, and increased inflammatory cell infiltration within the prostate stroma, indicating that stromal and tissue remodeling are more important determinants of age-associated tumor behavior in this cancer [175]. Collectively, these findings indicate that impaired immune surveillance and chronic inflammation represent shared consequences of aging. In contrast, the specific pathways linking aging to tumor progression and/or treatment resistance depend on the cellular composition and biological characteristics of each TME. Therefore, age-related biomarkers or therapeutic strategies useful in one cancer type should not be assumed to have the same prognostic or predictive value in another cancer without tumor-specific validation. A systematic comparison of these mechanisms across tumor types helps distinguish conserved features of immunosenescence from cancer-specific alterations and improves the clinical interpretation of age-related immune biomarkers.

The combination of ICIs with epigenetic modulators holds potential for counteracting age-related immunosenescence-associated therapeutic resistance. For instance, decitabine combined with anti-PD-1 therapy has been reported to enhance the expression of the AP-1 transcription factor JunD in CD8^+^ T cells, whose expression decreases after PD-1 blockade, thus preventing T cell senescence and promoting proliferation [176]. Likewise, the combination of anti-PD-1 with the subtype-selective HDAC inhibitor tucidinostat has been shown to decrease NF-κB signaling in CD8^+^ T cells and to attenuate senescence [177]. DNA methyltransferase inhibitors further reduce the production of pro-inflammatory cytokines, including IFN-γ, TNF-α, and IL-1β, thereby potentially limiting inflammation and immunosenescence [178]. Notably, some treatments also carry age-specific risks. For instance, anti-CD40/IL-2 treatment induces deadly cytokine storms in aged mice, characterized by excessive macrophage-mediated production of TNF, IL-6, and IFN-γ, leading to liver damage. Conversely, administration of the TNF antagonist etanercept preserves survival without affecting the antitumor effects of the treatment, suggesting that selective modulation of inflammatory toxicity improves the therapeutic window in older individuals [179].

The divergent effects of immunotherapies in young and old hosts partly reflect cell- or tissue-specific variations in the expression and function of immunosuppressive molecules. For example, PD-L1 inhibitors are less effective in aged mice, whereas PD-1 inhibitors retain comparable efficacy in young and old melanoma models. This discrepancy is explained by older DCs having higher levels of both PD-L1 and CD80. These molecules form heterodimers that neutralize the immunosuppressive effect of PD-L1 while maintaining the co-stimulatory function of CD80. In aged mice, PD-L1 blockade induces heterodimer formation and decreases co-stimulation, paradoxically boosting immunosuppression [180]. Given the growing proportion of older individuals among patients with cancer, clinical trials on this population are highly sought after. Although most of the Phase II trials are still ongoing or awaiting results, completed trials have shown encouraging safety and effectiveness results. Notably, multicenter trials have shown that older adults with relapsed/refractory large B-cell lymphoma (R/R LBCL) treated with CAR-T therapy achieve similar overall response rates, complete remission (CR), median overall survival, and progression-free survival comparable to those observed in younger patients [181].

In summary, although safety varies by tumor type, older individuals respond to immunotherapy similarly to their younger counterparts. Furthermore, whereas epigenetic medications increase effectiveness by lowering immunosenescence, IL-6 blocking improves ICI safety. Additionally, ICIs are further enhanced by adjuvant therapies, such as probiotics [10] and JAK inhibitors [182]. Nevertheless, clinical validation is still limited in older populations.

## 8. Conclusions and Perspectives

As the global population continues to age, the proportion of older patients affected by cancer is expected to increase substantially, making it essential to understand how aging affects the immune system, cancer development, and therapeutic responses. This review discusses immunosenescence as a multifactorial process initiated by genomic instability, telomere attrition, epigenetic alterations, the loss of proteostasis, altered intercellular communication, and chronic inflammation, all of which collectively contribute to the progressive decline of immune function during aging. Mitochondrial dysfunction is a relevant component of this network, as it affects immune cell metabolism, oxidative balance, and inflammatory signaling, and interacts with other age-related alterations. However, the extent to which mitochondrial dysfunction independently drives immunosenescence in aged, tumor-bearing hosts remains incompletely deciphered, as most mechanistic evidence has been obtained from other disease or experimental models. Additional studies in aged cancer models and older patients are therefore needed to define its specific contribution to tumor-associated immune dysfunction. When mitochondrial function is impaired in aging immune cells, altered metabolism and increased oxidative stress contribute to inflammaging and reduced immune surveillance. These changes also remodel the TME, making it more supportive of cancer growth and less responsive to therapy.

Current immunotherapies show mixed results in older patients. While some treatments work equally well across age groups, others are less effective due to age-related immune defects that arise from the combined effects of these hallmarks. Older patients also experience different patterns of side effects, which require careful management. Understanding these age-specific differences and the relative contribution of each mechanism is critical for improving cancer care in older adults.

Future research should focus on developing therapies that target multiple aspects of immunosenescence simultaneously. Promising approaches include restoring mitochondrial health, reducing chronic inflammation, eliminating senescent cells, and modulating epigenetic or proteostatic pathways. However, the therapeutic relevance of mitochondrial-targeted strategies should be established in aged, tumor-bearing models and older populations. Combining these strategies with existing immunotherapies could improve outcomes in older patients. Researchers also need better biomarkers to identify which older patients will benefit most from specific treatments and which are at the highest risk for side effects.

Several age-related gene- and long non-coding RNA (lncRNA)-based prognostic models have been proposed across cancer types, yet the molecular features retained in these signatures vary considerably among studies. Age-related gene signatures have been developed and externally evaluated in ovarian, pancreatic, and hepatocellular cancers, while distinct sets of aging- or senescence-related lncRNAs have been incorporated into prognostic models for hepatocellular [183], breast [184], and gastric cancers [185]. Such variability likely reflects differences in tumor biology, cohort characteristics, transcriptomic platforms, definitions of aging-related features, and feature-selection methods, limiting the comparability and generalizability of individual risk scores. Clinical translation therefore requires rigorous external validation in independent and clinically representative cohorts, with the assessment not only of discrimination but also of calibration and clinical utility. Harmonized definitions of aging-related molecular features, together with standardized scoring procedures and cut-off values, will be essential to improve model reproducibility, enable cross-study comparisons, and determine whether these signatures provide robust prognostic information across populations and clinical settings.

Clinical trials must include more older patients and consider biological age, not just chronological age. Treatments should be personalized to individual immune function rather than using a one-size-fits-all approach. Ultimately, addressing immunosenescence will require a more comprehensive approach encompassing multiple interconnected mechanisms rather than focusing on a single pathway. Translating these insights into clinically applicable strategies may enable safer and more effective cancer treatments for the growing population of older adults while preserving their quality of life.

## Data Availability

No new data were created or analyzed in this study. Data sharing is not applicable to this article.

## References

[1] Shevyrev D., Tereshchenko V., Berezina T.N., Rybtsov S. (2023). Hematopoietic stem cells and the immune system in development and aging. Int. J. Mol. Sci..

[2] Wang Q., Yuan Y., Liu J., Li C., Jiang X. (2024). The role of mitochondria in aging, cell death, and tumor immunity. Front. Immunol..

[3] Zhang F., Guo J., Yu S., Zheng Y., Duan M., Zhao L., Wang Y., Yang Z., Jiang X. (2024). Cellular senescence and metabolic reprogramming: Unraveling the intricate crosstalk in the immunosuppressive tumor microenvironment. Cancer Commun..

[4] Li X., Li C., Zhang W., Wang Y., Qian P., Huang H. (2023). Inflammation and aging: Signaling pathways and intervention therapies. Signal Transduct. Target Ther..

[5] Zarlashat Y., Ghaffar A., Guerra F., Picca A. (2025). Immunological landscape and molecular therapeutic targets of the tumor microenvironment in hepatocellular carcinoma. Int. J. Mol. Sci..

[6] Zarlashat Y., Picca A. (2026). Mitochondrial quality control and metabolic reprogramming in hepatocellular carcinoma: Implications for immunotherapy and treatment resistance. Cells.

[7] Li H., Jin W., Liu J., Zhou Y., Shan X., Zhang Y., Kou Y., Deng C., Jin C., Kuang J. (2025). Optimizing mitochondria function in immune cells: Implications for cancer immunotherapy. Trends Cancer.

[8] Zarlashat Y., Mushtaq H., Pham L., Abbas W., Sato K. (2024). Advancements in immunotherapeutic treatments for hepatocellular carcinoma: Potential of combination therapies. Int. J. Mol. Sci..

[9] Al-Danakh A., Safi M., Jian Y., Yang L., Zhu X., Chen Q., Yang K., Wang S., Zhang J., Yang D. (2024). Aging-related biomarker discovery in the era of immune checkpoint inhibitors for cancer patients. Front. Immunol..

[10] Yang H., Liu D., Qiu L., Wang R., Zhang C., Yu D., Zhong Q., Yuki N., Song Z., Zhu T. (2025). Reprogramming cellular senescence and aging clocks for advanced cancer immunotherapy. Mol. Cancer.

[11] Cho Y.H., Woo H.D., Jang Y., Porter V., Christensen S., Hamilton R.F., Chung H.W. (2015). The association of LINE-1 hypomethylation with age and centromere positive micronuclei in human lymphocytes. PLoS ONE.

[12] Zhao Y., Simon M., Seluanov A., Gorbunova V. (2023). DNA damage and repair in age-related inflammation. Nat. Rev. Immunol..

[13] Jia L., Wei Z., Luoqian J., Wang X., Huang C. (2025). Mitochondrial dysfunction in aging: Future therapies and precision medicine approaches. MedComm—Future Med..

[14] Needham B.L., Wang X., Carroll J.E., Barber S., Sánchez B.N., Seeman T.E., Roux A.V.D. (2019). Sociodemographic correlates of change in leukocyte telomere length during mid-to late-life: The Multi-Ethnic Study of Atherosclerosis. Psychoneuroendocrinology.

[15] Yousefzadeh M.J., Flores R.R., Zhu Y., Schmiechen Z.C., Brooks R.W., Trussoni C.E., Cui Y., Angelini L., Lee K.-A., McGowan S.J. (2021). An aged immune system drives senescence and ageing of solid organs. Nature.

[16] Lai T.P., Wright W.E., Shay J.W. (2018). Comparison of telomere length measurement methods. Philos. Trans. R Soc. Lond. B Biol. Sci..

[17] Maxwell F., McGlynn L.M., Muir H.C., Talwar D., Benzeval M., Robertson T., Roxburgh C.S., McMillan D.C., Horgan P.G., Shiels P.G. (2011). Telomere attrition and decreased fetuin-A levels indicate accelerated biological aging and are implicated in the pathogenesis of colorectal cancer. Clin. Cancer Res..

[18] Zhao J., Zhu Y., Uppal K., Tran V.T., Yu T., Lin J., Matsuguchi T., Blackburn E., Jones D., Lee E.T. (2014). Metabolic profiles of biological aging in American Indians: The strong heart family study. Aging.

[19] Kosmopoulos M., Chiriaco M., Stamatelopoulos K., Tsioufis C., Masci P.G., Kontogiannis C., Mengozzi A., Pugliese N.R., Taddei S., Virdis A. (2022). The relationship between telomere length and putative markers of vascular ageing: A systematic review and meta-analysis. Mech. Ageing Dev..

[20] Yang N.Y., Hsieh A.Y., Chen Z., Campbell A.R., Gadawska I., Kakkar F., Sauve L., Bitnun A., Brophy J., Murray M.C. (2024). Chronic and latent viral infections and leukocyte telomere length across the lifespan of female and male individuals living with or without HIV. Viruses.

[21] Cao D., Zhao J., Nguyan L.N., Nguyen L.N., Khanal S., Dang X., Schank M., Chand Thakuri B.K., Wu X.Y., Morrison Z.D. (2019). Disruption of telomere integrity and DNA repair machineries by KML001 induces T cell senescence, apoptosis, and cellular dysfunctions. Front. Immunol..

[22] Liang R., Tang Q., Chen J., Zhu L. (2024). Epigenetic clocks: Beyond biological age, using the past to predict the present and future. Aging Dis..

[23] Marttila S., Rajić S., Ciantar J., Mak J.K., Junttila I.S., Kummola L., Hägg S., Raitoharju E., Kananen L. (2025). Biological aging of different blood cell types. Geroscience.

[24] Mogilenko D.A., Shpynov O., Andhey P.S., Arthur L., Swain A., Esaulova E., Brioschi S., Shchukina I., Kerndl M., Bambouskova M. (2021). Comprehensive profiling of an aging immune system reveals clonal GZMK^+^ CD8^+^ T cells as conserved hallmark of inflammaging. Immunity.

[25] Lemus A.J., Tewelde E., Bhala R., Guan A., Xu A., Benayoun B.A. (2026). The interplay of epigenetic remodelling and transposon-mediated genomic instability in ageing and longevity. Open Biol..

[26] Kim C., Jin J., Ye Z., Jadhav R.R., Gustafson C.E., Hu B., Cao W., Tian L., Weyand C.M., Goronzy J.J. (2021). Histone deficiency and accelerated replication stress in T cell aging. J. Clin. Investig..

[27] Brunner S., Herndler-Brandstetter D., Arnold C.R., Wiegers G.J., Villunger A., Hackl M., Grillari J., Moreno-Villanueva M., Bürkle A., Grubeck-Loebenstein B. (2012). Upregulation of miR-24 is associated with a decreased DNA damage response upon etoposide treatment in highly differentiated CD8+ T cells sensitizing them to apoptotic cell death. Aging Cell.

[28] Ma F., Cao Y., Du H., Braikia F.Z., Zong L., Ollikainen N., Bayer M., Qiu X., Park B., Roy R. (2024). Three-dimensional chromatin reorganization regulates B cell development during ageing. Nat. Cell Biol..

[29] Yue Z., Nie L., Ji N., Sun Y., Zhu K., Zou H., Song X., Chen J., Wang Q. (2023). Hyperglycaemia aggravates periodontal inflamm-aging by promoting SETDB1-mediated LINE-1 de-repression in macrophages. J. Clin. Periodontol..

[30] Funk M.C., Gleixner J.G., Heigwer F., Vonficht D., Valentini E., Aydin Z., Tonin E., Del Prete S., Mahara S., Throm Y. (2023). Aged intestinal stem cells propagate cell-intrinsic sources of inflammaging in mice. Dev. Cell.

[31] Wang D., Chen K., Wang Z., Wu H., Li Y. (2024). Research progress on interferon and cellular senescence. FASEB J..

[32] Rasa S.M.M., Annunziata F., Krepelova A., Nunna S., Omrani O., Gebert N., Adam L., Käppel S., Höhn S., Donati G. (2022). Inflammaging is driven by upregulation of innate immune receptors and systemic interferon signaling and is ameliorated by dietary restriction. Cell Rep..

[33] DelaO-Escamilla A., Khalil S., Galadari H., Guida S. (2025). Epigenetic clocks in skin aging: From exposome drivers to biomarkers and therapeutic interventions. Clin. Cosmet. Investig. Dermatol..

[34] Oltmanns C., Liu Z., Mischke J., Tauwaldt J., Mekonnen Y.A., Urbanek-Quaing M., Debarry J., Maasoumy B., Wedemeyer H., Kraft A.R. (2023). Reverse inflammaging: Long-term effects of HCV cure on biological age. J. Hepatol..

[35] Gindin Y., Gaggar A., Lok A.S., Janssen H.L., Ferrari C., Subramanian G.M., Jiang Z., Masur H., Emmanuel B., Poonia B. (2021). DNA methylation and immune cell markers demonstrate evidence of accelerated aging in patients with chronic hepatitis B virus or hepatitis C virus, with or without human immunodeficienct virus co-infection. Clin. Infect. Dis..

[36] Durso D., Silveira-Nunes G., Coelho M., Camatta G., Ventura L., Nascimento L., Caixeta F., Cunha E., Castelo-Branco A., Fonseca D.M.d. (2022). Living in endemic area for infectious diseases accelerates epigenetic age. Mech. Ageing Dev..

[37] Conole E.L., Stevenson A.J., Muñoz Maniega S., Harris S.E., Green C., Valdés Hernández M.d.C., Harris M.A., Bastin M.E., Wardlaw J.M., Deary I.J. (2021). DNA methylation and protein markers of chronic inflammation and their associations with brain and cognitive aging. Neurology.

[38] Christofidou P., Bell C.G. (2025). The predictive power of profiling the DNA methylome in human health and disease. Epigenomics.

[39] Bradburn S., McPhee J., Bagley L., Carroll M., Slevin M., Al-Shanti N., Barnouin Y., Hogrel J.-Y., Pääsuke M., Gapeyeva H. (2018). Dysregulation of CXC motif ligand 10 during aging and association with cognitive performance. Neurobiol. Aging.

[40] Cheng H., Xuan H., Green C.D., Han Y., Sun N., Shen H., McDermott J., Bennett D.A., Lan F., Han J.-D.J. (2018). Repression of human and mouse brain inflammaging transcriptome by broad gene-body histone hyperacetylation. Proc. Natl. Acad. Sci. USA.

[41] Hipp M.S., Kasturi P., Hartl F.U. (2019). The proteostasis network and its decline in ageing. Nat. Rev. Mol. Cell Biol..

[42] Di Conza G., Ho P.-C., Cubillos-Ruiz J.R., Huang S.C.-C. (2023). Control of immune cell function by the unfolded protein response. Nat. Rev. Immunol..

[43] Jia Y., Li Z., Huang P., Wang Y., Yang B. (2025). Mechanisms and therapeutic perspectives of mitochondrial dysfunction of macrophages in periodontitis. Front. Cell. Infect. Microbiol..

[44] Chen W., Xiao W., Liu X., Yuan P., Zhang S., Wang Y., Wu W. (2022). Pharmacological manipulation of macrophage autophagy effectively rejuvenates the regenerative potential of biodegrading vascular graft in aging body. Bioact. Mater..

[45] Plaza-Zabala A., Sierra-Torre V., Sierra A. (2017). Autophagy and microglia: Novel partners in neurodegeneration and aging. Int. J. Mol. Sci..

[46] Ye F., Wei C., Wu A. (2024). The potential mechanism of mitochondrial homeostasis in postoperative neurocognitive disorders: An in-depth review. Ann. Med..

[47] Lindsey L.P., Daphney C.M., Oppong-Damoah A., Uchakin P.N., Abney S.E., Uchakina O.N., Khusial R.D., Akil A., Murnane K.S. (2019). The cannabinoid receptor 2 agonist, β-caryophyllene, improves working memory and reduces circulating levels of specific proinflammatory cytokines in aged male mice. Behav. Brain Res..

[48] Wendimu M.Y., Hooks S.B. (2022). Microglia phenotypes in aging and neurodegenerative diseases. Cells.

[49] Ritzel R.M., Wu J. (2023). Functional phenotyping of microglia highlights the dark relationship between chronic traumatic brain injury and normal age-related pathology. Neural Reg. Res..

[50] Berglund R., Cheng Y., Piket E., Adzemovic M.Z., Zeitelhofer M., Olsson T., Guerreiro-Cacais A.O., Jagodic M. (2024). The aging mouse CNS is protected by an autophagy-dependent microglia population promoted by IL-34. Nat. Commun..

[51] Jalloh A., Flowers A., Hudson C., Chaput D., Guergues J., Stevens S.M., Bickford P.C. (2021). Polyphenol supplementation reverses Age-Related changes in microglial signaling cascades. Int. J. Mol. Sci..

[52] Runyan C.E., Welch L.C., Lecuona E., Shigemura M., Amarelle L., Abdala-Valencia H., Joshi N., Lu Z., Nam K., Markov N.S. (2020). Impaired phagocytic function in CX3CR1^+^ tissue-resident skeletal muscle macrophages prevents muscle recovery after influenza A virus-induced pneumonia in old mice. Aging Cell.

[53] Frankowska N., Lisowska K., Witkowski J.M. (2022). Proteolysis dysfunction in the process of aging and age-related diseases. Front. Aging.

[54] Gohlke S., Mishto M., Textoris-Taube K., Keller C., Giannini C., Vasuri F., Capizzi E., D’Errico-Grigioni A., Kloetzel P.-M., Dahlmann B. (2014). Molecular alterations in proteasomes of rat liver during aging result in altered proteolytic activities. Age.

[55] Rana P.S., Ignatz-Hoover J.J., Driscoll J.J. (2023). Targeting proteasomes and the MHC Class I antigen presentation machinery to treat cancer, infections and age-related diseases. Cancers.

[56] De Benedictis G., Rose G., Carrieri G., De Luca M., Falcone E., Passarino G., Bonafè M., Monti D., Baggio G., Bertolini S. (1999). Mitochondrial DNA inherited variants are associated with successful aging and longevity in humans. FASEB J..

[57] Gonzalez B., Tare A., Ryu S., Johnson S.C., Atzmon G., Barzilai N., Kaeberlein M., Suh Y. (2023). High-throughput sequencing analysis of nuclear-encoded mitochondrial genes reveals a genetic signature of human longevity. Geroscience.

[58] Gupta R., Durham T.J., Chau G., Kanai M., Uddin M.M., Lu W., Argentieri M.A., Karczewski K.J., Howrigan D., Natarajan P. (2026). Mechanism of age-related accumulation of mtDNA mutations in human blood. Nature.

[59] Narendra D.P., Youle R.J. (2024). The role of PINK1–Parkin in mitochondrial quality control. Nat. Cell Biol..

[60] Higuchi-Sanabria R., Frankino P.A., Paul J.W., Tronnes S.U., Dillin A. (2018). A futile battle? Protein quality control and the stress of aging. Dev. Cell.

[61] Lewis L., Valvi D., Gedaly R., Marti F. (2025). Mitochondrial unfolded protein response in regulatory T cell function: A protective mechanism in immune aging. Front. Immunol..

[62] Picca A., Lezza A.M. (2015). Regulation of mitochondrial biogenesis through TFAM-mitochondrial DNA interactions: Useful insights from aging and calorie restriction studies. Mitochondrion.

[63] Xu X., Pang Y., Fan X. (2025). Mitochondria in oxidative stress, inflammation and aging: From mechanisms to therapeutic advances. Signal Transduct. Target. Ther..

[64] Li F., Feng Y., Yin Z., Wang Y. (2025). Mitochondrial metabolism in T-cell exhaustion. Int. J. Mol. Sci..

[65] Mittelbrunn M., Kroemer G. (2021). Hallmarks of T cell aging. Nat. Immunol..

[66] Headley C.A., Gautam S., Olmo-Fontanez A., Garcia-Vilanova A., Dwivedi V., Schami A., Weintraub S., Tsao P.S., Torrelles J.B., Turner J. (2024). Mitochondrial transplantation promotes protective effector and memory CD4+ T cell response during Mycobacterium tuberculosis infection and diminishes exhaustion and senescence in elderly CD4+ T cells. Adv. Sci..

[67] Chen Y., Ye Y., Krauß P.-L., Löwe P., Pfeiffenberger M., Damerau A., Ehlers L., Buttgereit T., Hoff P., Buttgereit F. (2022). Age-related increase of mitochondrial content in human memory CD4+ T cells contributes to ROS-mediated increased expression of proinflammatory cytokines. Front. Immunol..

[68] Chougnet C.A., Thacker R.I., Shehata H.M., Hennies C.M., Lehn M.A., Lages C.S., Janssen E.M. (2015). Loss of phagocytic and antigen cross-presenting capacity in aging dendritic cells is associated with mitochondrial dysfunction. J. Immunol..

[69] Panel M., Ghaleh B., Morin D. (2018). Mitochondria and aging: A role for the mitochondrial transition pore?. Aging Cell.

[70] Ryu S., Sidorov S., Ravussin E., Artyomov M., Iwasaki A., Wang A., Dixit V.D. (2022). The matricellular protein SPARC induces inflammatory interferon-response in macrophages during aging. Immunity.

[71] Ye B., Pei Y., Wang L., Meng D., Zhang Y., Zou S., Li H., Liu J., Xie Z., Tian C. (2024). NAD+ supplementation prevents STING-induced senescence in CD8+ T cells by improving mitochondrial homeostasis. J. Cell. Biochem..

[72] Qiu X., Guo H., Yang J., Ji Y., Wu C.-S., Chen X. (2018). Down-regulation of guanylate binding protein 1 causes mitochondrial dysfunction and cellular senescence in macrophages. Sci. Rep..

[73] Sawoo R., Dey R., Ghosh R., Bishayi B. (2021). TLR4 and TNFR1 blockade dampen M1 macrophage activation and shifts them towards an M2 phenotype. Immunol. Res..

[74] Xie Z., Lin M., Xing B., Wang H., Zhang H., Cai Z., Mei X., Zhu Z.-J. (2025). Citrulline regulates macrophage metabolism and inflammation to counter aging in mice. Sci. Adv..

[75] Cui Z., Zhao X., Amevor F.K., Du X., Wang Y., Li D., Shu G., Tian Y., Zhao X. (2022). Therapeutic application of quercetin in aging-related diseases: SIRT1 as a potential mechanism. Front. Immunol..

[76] Liu Y., Yang C., Zhang J., Ihsan A., Ares I., Martínez M., Lopez-Torres B., Martínez-Larrañaga M.-R., Wang X., Anadón A. (2024). Recent progress in adverse events of carboxylic acid non-steroidal anti-inflammatory drugs (CBA-NSAIDs) and their association with the metabolism: The consequences on mitochondrial dysfunction and oxidative stress, and prevention with natural plant extracts. Expert Opin. Drug Metab. Toxicol..

[77] Zhao J., Bu F., Wu H., He J., Liu J. (2026). Neuroinflammation and NeuroHIV: Understanding the role of HIV-1 related factors in microglial activation. Transl. Psychiatry.

[78] Hussain M., Chu X., Sahbaz B.D., Gray S., Pekhale K., Park J.-H., Croteau D.L., Bohr V.A. (2023). Mitochondrial OGG1 expression reduces age-associated neuroinflammation by regulating cytosolic mitochondrial DNA. Free Radic. Biol. Med..

[79] Miller K.N., Dasgupta N., Liu T., Adams P.D., Vizioli M.G. (2021). Cytoplasmic chromatin fragments—From mechanisms to therapeutic potential. Elife.

[80] Teng C., Wei J., Zhu Y., Chen A., Chen X. (2026). Mitochondrial dysfunction in neonatal brain injury: From molecular mechanisms to therapeutic interventions. J. Transl. Med..

[81] Yousef H., Czupalla C.J., Lee D., Chen M.B., Burke A.N., Zera K.A., Zandstra J., Berber E., Lehallier B., Mathur V. (2019). Aged blood impairs hippocampal neural precursor activity and activates microglia via brain endothelial cell VCAM1. Nat. Med..

[82] Balter L.J., Higgs S., Aldred S., Bosch J.A., Raymond J.E. (2019). Inflammation mediates body weight and ageing effects on psychomotor slowing. Sci. Rep..

[83] Wenzek C., Boelen A., Westendorf A.M., Engel D.R., Moeller L.C., Führer D. (2022). The interplay of thyroid hormones and the immune system–where we stand and why we need to know about it. Eur. J. Endocrinol..

[84] Irina L. (2015). Immune-neuroendocrine interactions involving thymus and pineal gland in stem cell therapy of age-related diseases. Immunol. Endocr. Metab. Agents Med. Chem..

[85] Cui K., Tang X., Hu A., Fan M., Wu P., Lu X., Lin J., Yang F., Zhao X., Huang J. (2023). Therapeutic benefit of melatonin in choroidal neovascularization during aging through the regulation of senescent macrophage/microglia polarization. Investig. Ophthalmol. Vis. Sci..

[86] Mazzoccoli G., Inglese M., De Cata A., Carughi S., Dagostino M.P., Marzulli N., Damato M., Grilli M., Giuliani F., Greco A. (2011). Neuroendocrine–immune interactions in healthy aging. Geriatr. Gerontol. Int..

[87] Koelman L., Pivovarova-Ramich O., Pfeiffer A.F., Grune T., Aleksandrova K. (2019). Cytokines for evaluation of chronic inflammatory status in ageing research: Reliability and phenotypic characterisation. Immun. Ageing.

[88] Laskow T., Langdon J., Sepehri S., Davalos-Bichara M., Varadhan R., Walston J. (2024). Soluble TNFR1 has greater reproducibility than IL-6 for the assessment of chronic inflammation in older adults: The case for a new inflammatory marker in aging. Geroscience.

[89] Pinti M., Cevenini E., Nasi M., De Biasi S., Salvioli S., Monti D., Benatti S., Gibellini L., Cotichini R., Stazi M.A. (2014). Circulating mitochondrial DNA increases with age and is a familiar trait: Implications for “inflamm-aging”. Eur. J. Immunol..

[90] Seicol B.J., Lin S., Xie R. (2022). Age-related hearing loss is accompanied by chronic inflammation in the cochlea and the cochlear nucleus. Front. Aging Neurosci..

[91] Bruno M.E., Mukherjee S., Powell W.L., Mori S.F., Wallace F.K., Balasuriya B.K., Su L.C., Stromberg A.J., Cohen D.A., Starr M.E. (2022). Accumulation of γδ T cells in visceral fat with aging promotes chronic inflammation. Geroscience.

[92] Ruan P., Yang M., Lv X., Shen K., Chen Y., Li H., Zhao D., Huang J., Xiao Y., Peng W. (2024). Metabolic shifts during coffee consumption refresh the immune response: Insight from comprehensive multiomics analysis. MedComm.

[93] Robbins P.D., Jurk D., Khosla S., Kirkland J.L., LeBrasseur N.K., Miller J.D., Passos J.F., Pignolo R.J., Tchkonia T., Niedernhofer L.J. (2021). Senolytic drugs: Reducing senescent cell viability to extend health span. Annu. Rev. Pharmacol. Toxicol..

[94] Wong C.P., Magnusson K.R., Sharpton T.J., Ho E. (2021). Effects of zinc status on age-related T cell dysfunction and chronic inflammation. Biometals.

[95] Morita Y., Jounai K., Sakamoto A., Tomita Y., Sugihara Y., Suzuki H., Ohshio K., Otake M., Fujiwara D., Kanauchi O. (2018). Long-term intake of Lactobacillus paracasei KW3110 prevents age-related chronic inflammation and retinal cell loss in physiologically aged mice. Aging.

[96] Magni O., Arnaoutis G., Panagiotakos D. (2025). The impact of exercise on chronic systemic inflammation: A systematic review and meta–meta-analysis. Sport Sci. Health.

[97] Woolbright B.L., Xuan H., Ahmed I., Rajendran G., Abbott E., Dennis K., Zhong C., Umar S., Taylor J.A. (2024). Aging induces changes in cancer formation and microbial content in a murine model of bladder cancer. Geroscience.

[98] Li M., Liu J.-X., Ma B., Liu J.-Y., Chen J., Jin F., Hu C.-H., Xu H.-K., Zheng C.-X., Hou R. (2024). A Senescence-Associated secretory phenotype of bone marrow mesenchymal stem cells inhibits the viability of breast cancer cells. Stem Cell Rev. Rep..

[99] Nakajima Y., Chamoto K., Oura T., Honjo T. (2021). Critical role of the CD44lowCD62Llow CD8+ T cell subset in restoring antitumor immunity in aged mice. Proc. Natl. Acad. Sci. USA.

[100] Costa A.C., Santos J.M., da Costa R.M.G., Medeiros R. (2021). Impact of immune cells on the hallmarks of cancer: A literature review. Crit. Rev. Oncol./Hematol..

[101] Kim C.M., Lee J.B., Shin S.J., Ahn J.B., Lee M., Kim H.S. (2022). The efficacy of immune checkpoint inhibitors in elderly patients: A meta-analysis and meta-regression. ESMO Open.

[102] Esen B.H., Bektas S.N., Topcu U., Köylü B., Kuvvet F.B.B., Bahat G., Selçukbiricik F. (2025). Immune-related adverse events in older adults receiving immune checkpoint inhibitors: A comprehensive analysis of the Food and Drug Administration Adverse Event Reporting System. Age Ageing.

[103] Gesierich A., Demeter E., Schilling B., Deschler-Baier B. (2026). More than Age: Impact of comorbidity and polypharmacy on immune-related adverse events, treatment discontinuation, and toxicity management in older patients receiving immune checkpoint inhibitors. J. Geriatr. Oncol..

[104] Tan L., Xiaohalati X., Liu F., Liu J., Fu H., Zhang Y., Gao J., Tao K., Wang G., Wang L. (2023). Identification of aging and young subtypes for predicting colorectal cancer prognosis and immunotherapy responses. Int. J. Mol. Sci..

[105] He Z., Chen M., Li Q., Luo Z., Li X. (2024). Multi-omics and tumor immune microenvironment characterization of a prognostic model based on aging-related genes in melanoma. Am. J. Cancer Res..

[106] Zhang W., Li Y., Lyu J., Shi F., Kong Y., Sheng C., Wang S., Wang Q. (2022). An aging-related signature predicts favorable outcome and immunogenicity in lung adenocarcinoma. Cancer Sci..

[107] Mu J., Gong J., Shi M., Zhang Y. (2023). Analysis and validation of aging-related genes in prognosis and immune function of glioblastoma. BMC Med. Genom..

[108] Wu Y., Li X. (2023). Senescence gene expression in clear cell renal cell carcinoma: Role of tumor immune microenvironment and senescence-associated survival prediction. Medicine.

[109] Hong T., Su W., Pan Y., Tian C., Lei G. (2022). Aging-related features predict prognosis and immunotherapy efficacy in hepatocellular carcinoma. Front. Immunol..

[110] Chen F., Gong X., Xia M., Yu F., Wu J., Yu C., Li J. (2022). The aging-related prognostic signature reveals the landscape of the tumor immune microenvironment in head and neck squamous cell carcinoma. Front. Oncol..

[111] Gao Q., Shi Y., Sun Y., Zhou S., Liu Z., Sun X., Di X. (2023). Identification and verification of aging-related lncRNAs for prognosis prediction and immune microenvironment in patients with head and neck squamous carcinoma. Oncol. Res..

[112] Liu Q., Bao H., Zhang S., Song T., Li C., Sun G., Sun X., Fu T., Wang Y., Liang P. (2023). Identification of a cellular senescence-related-lncRNA (SRlncRNA) signature to predict the overall survival of glioma patients and the tumor immune microenvironment. Front. Genet..

[113] Huang E., Ma T., Zhou J., Ma N., Yang W., Liu C., Hou Z., Chen S., Zong Z., Zeng B. (2022). A novel senescence-associated LncRNA signature predicts the prognosis and tumor microenvironment of patients with colorectal cancer: A bioinformatics analysis. J. Gastrointest. Oncol..

[114] Zhou Z., Wei J., Kuang L., Zhang K., Liu Y., He Z., Li L., Lu B. (2022). Characterization of aging cancer-associated fibroblasts draws implications in prognosis and immunotherapy response in low-grade gliomas. Front. Genet..

[115] Zarlashat Y., Abbas S., Ghaffar A. (2024). Hepatocellular carcinoma: Beyond the border of advanced stage therapy. Cancers.

[116] Wang Z., Zhao Y., Zhang L. (2025). Emerging trends and hot topics in the application of multi-omics in drug discovery: A bibliometric and visualized study. Curr. Pharm. Anal..

[117] Zöphel D., Angenendt A., Kaschek L., Ravichandran K., Hof C., Janku S., Hoth M., Lis A. (2022). Faster cytotoxicity with age: Increased perforin and granzyme levels in cytotoxic CD8+ T cells boost cancer cell elimination. Aging Cell.

[118] Jackaman C., Gardner J.K., Tomay F., Spowart J., Crabb H., Dye D.E., Fox S., Proksch S., Metharom P., Dhaliwal S.S. (2019). CD8+ cytotoxic T cell responses to dominant tumor-associated antigens are profoundly weakened by aging yet subdominant responses retain functionality and expand in response to chemotherapy. Oncoimmunology.

[119] Vaena S., Chakraborty P., Lee H.G., Janneh A.H., Kassir M.F., Beeson G., Hedley Z., Yalcinkaya A., Sofi M.H., Li H. (2021). Aging-dependent mitochondrial dysfunction mediated by ceramide signaling inhibits antitumor T cell response. Cell Rep..

[120] Chen H.C., Eling N., Martinez-Jimenez C.P., O’Brien L.M., Carbonaro V., Marioni J.C., Odom D.T., de la Roche M. (2019). IL-7-dependent compositional changes within the γδ T cell pool in lymph nodes during ageing lead to an unbalanced anti-tumour response. EMBO Rep..

[121] Wang Y., Liang M., Mao Y., Zhu W., Shen X., Guan W. (2026). Aging modulation of the immune system and immunotherapy efficacy in cancer. Front. Immunol..

[122] Sharma S., Dominguez A.L., Lustgarten J. (2006). Aging affect the anti-tumor potential of dendritic cell vaccination, but it can be overcome by co-stimulation with anti-OX40 or anti-4-1BB. Exp. Gerontol..

[123] Sohrabi S., Alipour S., Ghahramanipour Z., Masoumi J., Baradaran B. (2024). STAT signaling pathways in immune cells and their associated mechanisms in cancer pathogenesis. BioImpacts.

[124] Kakuda T., Suzuki J., Matsuoka Y., Kikugawa T., Saika T., Yamashita M. (2023). Senescent CD8+ T cells acquire NK cell-like innate functions to promote antitumor immunity. Cancer Sci..

[125] Leibovici J., Itzhaki O., Kaptzan T., Skutelsky E., Sinai J., Michowitz M., Asfur R., Siegal A., Huszar M., Schiby G. (2009). Designing ageing conditions in tumour microenvironment—A new possible modality for cancer treatment. Mech. Ageing Dev..

[126] Shireman J.M., Gonugunta N., Zhao L., Pattnaik A., Distler E., Her S., Wang X., Das R., Galipeau J., Dey M. (2023). GM-CSF and IL-7 fusion cytokine engineered tumor vaccine generates long-term Th-17 memory cells and increases overall survival in aged syngeneic mouse models of glioblastoma. Aging Cell.

[127] Ly L.V., Baghat A., Versluis M., Jordanova E.S., Luyten G.P., van Rooijen N., van Hall T., van der Velden P.A., Jager M.J. (2010). In aged mice, outgrowth of intraocular melanoma depends on proangiogenic M2-type macrophages. J. Immunol..

[128] Haston S., Gonzalez-Gualda E., Morsli S., Ge J., Reen V., Calderwood A., Moutsopoulos I., Panousopoulos L., Deletic P., Carreno G. (2023). Clearance of senescent macrophages ameliorates tumorigenesis in KRAS-driven lung cancer. Cancer Cell.

[129] Mittmann L.A., Haring F., Schaubächer J.B., Hennel R., Smiljanov B., Zuchtriegel G., Canis M., Gires O., Krombach F., Holdt L. (2021). Uncoupled biological and chronological aging of neutrophils in cancer promotes tumor progression. J. Immunother. Cancer.

[130] Balliet R.M., Capparelli C., Guido C., Pestell T.G., Martinez-Outschoorn U.E., Lin Z., Whitaker-Menezes D., Chiavarina B., Pestell R.G., Howell A. (2011). Mitochondrial oxidative stress in cancer-associated fibroblasts drives lactate production, promoting breast cancer tumor growth: Understanding the aging and cancer connection. Cell Cycle.

[131] Zabransky D.J., Chhabra Y., Fane M.E., Kartalia E., Leatherman J.M., Hüser L., Zimmerman J.W., Delitto D., Han S., Armstrong T.D. (2024). Fibroblasts in the aged pancreas drive pancreatic cancer progression. Cancer Res..

[132] Liu Y., Pan J., Pan X., Wu L., Bian J., Lin Z., Xue M., Su T., Lai S., Chen F. (2019). Klotho-mediated targeting of CCL 2 suppresses the induction of colorectal cancer progression by stromal cell senescent microenvironments. Mol. Oncol..

[133] Li Y., Tazawa H., Nagai Y., Fujita S., Okura T., Shoji R., Yamada M., Kikuchi S., Kuroda S., Ohara T. (2024). Senescent fibroblasts potentiate peritoneal metastasis of Diffuse-type gastric cancer cells via IL-8–mediated crosstalk. Anticancer Res..

[134] Guo Y., Ayers J.L., Carter K.T., Wang T., Maden S.K., Edmond D., Newcomb P.P., Li C., Ulrich C., Yu M. (2019). Senescence-associated tissue microenvironment promotes colon cancer formation through the secretory factor GDF15. Aging Cell.

[135] Falcone I., Conciatori F., Bazzichetto C., Ferretti G., Cognetti F., Ciuffreda L., Milella M. (2020). Tumor microenvironment: Implications in melanoma resistance to targeted therapy and immunotherapy. Cancers.

[136] Liu X., Si F., Bagley D., Ma F., Zhang Y., Tao Y., Shaw E., Peng G. (2022). Blockades of effector T cell senescence and exhaustion synergistically enhance antitumor immunity and immunotherapy. J. Immunother. Cancer.

[137] Liu X., Hartman C.L., Li L., Albert C.J., Si F., Gao A., Huang L., Zhao Y., Lin W., Hsueh E.C. (2021). Reprogramming lipid metabolism prevents effector T cell senescence and enhances tumor immunotherapy. Sci. Transl. Med..

[138] Chen A.C., Jaiswal S., Martinez D., Yerinde C., Ji K., Miranda V., Fung M.E., Weiss S.A., Zschummel M., Taguchi K. (2024). The aged tumor microenvironment limits T cell control of cancer. Nat. Immunol..

[139] Ludwig L.S., Lareau C.A. (2023). Concomitant sequencing of accessible chromatin and mitochondrial genomes in single cells using mtscATAC-seq. Methods Mol. Biol..

[140] Hui K., Dong C., Hu C., Li J., Yan D., Jiang X. (2024). VEGFR affects miR-3200-3p-mediated regulatory T cell senescence in tumour-derived exosomes in non-small cell lung cancer. Funct. Integr. Genom..

[141] Gao A., Liu X., Lin W., Wang J., Wang S., Si F., Huang L., Zhao Y., Sun Y., Peng G. (2021). Tumor-derived ILT4 induces T cell senescence and suppresses tumor immunity. J. Immunother. Cancer.

[142] Grassi F., De Ponte Conti B. (2021). The P2X7 receptor in tumor immunity. Front. Cell Dev. Biol..

[143] Lee-Chang C., Bodogai M., Moritoh K., Chen X., Wersto R., Sen R., Young H.A., Croft M., Ferrucci L., Biragyn A. (2016). Aging converts innate B1a cells into potent CD8+ T cell inducers. J. Immunol..

[144] Tomihara K., Shin T., Hurez V.J., Yagita H., Pardoll D.M., Zhang B., Curiel T.J., Shin T. (2012). Aging-associated B7-DC+ B cells enhance anti-tumor immunity via Th1 and Th17 induction. Aging Cell.

[145] Ramos M.J., Lui A.J., Hollern D.P. (2023). The evolving landscape of B cells in cancer metastasis. Cancer Res..

[146] Göçer P., Gürer Ü.S., Erten N., Palandüz Ş., Rayaman E., Akarsu B., Karan A., Çevikbaş A. (2005). Comparison of polymorphonuclear leukocyte functions in elderly patients and healthy young volunteers. Med. Princ. Pract..

[147] Yang C., Wang Z., Li L., Zhang Z., Jin X., Wu P., Sun S., Pan J., Su K., Jia F. (2021). Aged neutrophils form mitochondria-dependent vital NETs to promote breast cancer lung metastasis. J. Immunother. Cancer.

[148] Guo F., Long L., Wang J., Wang Y., Liu Y., Wang L., Luo F. (2019). Insights on CXC chemokine receptor 2 in breast cancer: An emerging target for oncotherapy. Oncol. Lett..

[149] Bodac A., Mayet A., Rana S., Pascual J., Bowler A.D., Roh V., Fournier N., Craciun L., Demetter P., Radtke F. (2023). Bcl-xL targeting eliminates ageing tumor-promoting neutrophils and inhibits lung tumor growth. EMBO Mol. Med..

[150] Rodrigues-Santos P., López-Sejas N., Almeida J.S., Ruzičková L., Couceiro P., Alves V., Campos C., Alonso C., Tarazona R., Freitas-Tavares P. (2018). Effect of age on NK cell compartment in chronic myeloid leukemia patients treated with tyrosine kinase inhibitors. Front. Immunol..

[151] Sousa S., Brion R., Lintunen M., Kronqvist P., Sandholm J., Mönkkönen J., Kellokumpu-Lehtinen P.-L., Lauttia S., Tynninen O., Joensuu H. (2015). Human breast cancer cells educate macrophages toward the M2 activation status. Breast Cancer Res..

[152] Chen M., Su Z., Xue J. (2025). Targeting T-cell aging to remodel the aging immune system and revitalize geriatric immunotherapy. Aging Dis..

[153] Wang T., Liu C., Hu X., Yang N., Qiu C. (2025). Senescent macrophages in cancer: Roles in tumor progression and treatment opportunities. Cancer Biol. Med..

[154] Duong L., Radley-Crabb H.G., Gardner J.K., Tomay F., Dye D.E., Grounds M.D., Pixley F.J., Nelson D.J., Jackaman C. (2018). Macrophage depletion in elderly mice improves response to tumor immunotherapy, increases anti-tumor T cell activity and reduces treatment-induced cachexia. Front. Genet..

[155] Ontiveros C.O., Murray C.E., Crossland G., Curiel T.J. (2023). Considerations and approaches for cancer immunotherapy in the aging host. Cancer Immunol. Res..

[156] Kubo T., Ichihara E., Harada D., Inoue K., Fujiwara K., Hosokawa S., Kishino D., Kawai H., Ochi N., Oda N. (2023). Efficacy of immune checkpoint inhibitor monotherapy in elderly patients with non-small-cell lung cancer. Respir. Investig..

[157] Sattar J., Kartolo A., Hopman W.M., Lakoff J.M., Baetz T. (2019). The efficacy and toxicity of immune checkpoint inhibitors in a real-world older patient population. J. Geriatr. Oncol..

[158] Contieri R., Grajales V., Tan W.S., Martini A., Sood A., Hensley P., Bree K., Lobo N., Nogueras-Gonzalez G.M., Guo C.C. (2024). Impact of age > 70 years on oncological outcomes in patients with non-muscle-invasive bladder cancer treated with Bacillus Calmette–Guérin. BJU Int..

[159] Dolan M., Libby K.A., Ringel A.E., van Galen P., McAllister S.S. (2025). Ageing, Immune Fitness and Cancer. Nat. Rev. Cancer.

[160] Baldini C., Romano P.M., Voisin A.-L., Danlos F.-X., Champiat S., Laghouati S., Kfoury M., Vincent H., Postel-Vinay S., Varga A. (2020). Impact of aging on immune-related adverse events generated by anti–programmed death (ligand) PD-(L) 1 therapies. Eur. J. Cancer.

[161] Nemoto Y., Ishihara H., Nakamura K., Tachibana H., Fukuda H., Yoshida K., Kobayashi H., Iizuka J., Shimmura H., Hashimoto Y. (2022). Efficacy and safety of immune checkpoint inhibitors in elderly patients with metastatic renal cell carcinoma. Int. Urol. Nephrol..

[162] Zhou H., Cai L.-L., Lin Y.-F., Ma J.-J. (2024). Toxicity profile of camrelizumab-based immunotherapy in older adults with advanced cancer. Sci. Rep..

[163] Wang N., Zheng L., Li M., Hou X., Zhang B., Chen J., Li S., Chen L. (2023). Clinical efficacy and safety of individualized pembrolizumab administration based on pharmacokinetic in advanced non-small cell lung cancer: A prospective exploratory clinical trial. Lung Cancer.

[164] Schulz G.B., Rodler S., Szabados B., Graser A., Buchner A., Stief C., Casuscelli J. (2020). Safety, efficacy and prognostic impact of immune checkpoint inhibitors in older patients with genitourinary cancers. J. Geriatr. Oncol..

[165] Paderi A., Fancelli S., Caliman E., Pillozzi S., Gambale E., Mela M.M., Doni L., Mazzoni F., Antonuzzo L. (2021). Safety of immune checkpoint inhibitors in elderly patients: An observational study. Curr. Oncol..

[166] Wieczorek E., Garstka M.A. (2021). Recurrent bladder cancer in aging societies: Importance of major histocompatibility complex class I antigen presentation. Int. J. Cancer.

[167] Salminen A. (2024). The role of the immunosuppressive PD-1/PD-L1 checkpoint pathway in the aging process and age-related diseases. J. Mol. Med..

[168] Hou J., Xie S., Gao J., Jiang T., Zhu E., Yang X., Jin Z., Long H., Zhang A., Yang F. (2024). NK cell transfer overcomes resistance to PD-(L) 1 therapy in aged mice. Exp. Hematol. Oncol..

[169] Sitnikova S.I., Walker J.A., Prickett L.B., Morrow M., Valge-Archer V.E., Robinson M.J., Wilkinson R.W., Dovedi S.J. (2023). Age-induced changes in anti-tumor immunity alter the tumor immune infiltrate and impact response to immuno-oncology treatments. Front. Immunol..

[170] Dominguez A.L., Lustgarten J. (2008). Implications of aging and self-tolerance on the generation of immune and antitumor immune responses. Cancer Res..

[171] Zhang J., Wang S., Liu H., Luo L., You J. (2026). Arginine competition in tumor microenvironment: A potential target for cancer therapy. Med. Res. Rev..

[172] Sceneay J., Goreczny G.J., Wilson K., Morrow S., DeCristo M.J., Ubellacker J.M., Qin Y., Laszewski T., Stover D.G., Barrera V. (2019). Interferon signaling is diminished with age and is associated with immune checkpoint blockade efficacy in triple-negative breast cancer. Cancer Discov..

[173] Zhang M., Di Martino J.S., Bowman R.L., Campbell N.R., Baksh S.C., Simon-Vermot T., Kim I.S., Haldeman P., Mondal C., Yong-Gonzales V. (2018). Adipocyte-derived lipids mediate melanoma progression via FATP proteins. Cancer Discov..

[174] Provenzano P.P., Eliceiri K.W., Campbell J.M., Inman D.R., White J.G., Keely P.J. (2006). Collagen reorganization at the tumor-stromal interface facilitates local invasion. BMC Med..

[175] Bianchi-Frias D., Vakar-Lopez F., Coleman I.M., Plymate S.R., Reed M.J., Nelson P.S. (2010). The effects of aging on the molecular and cellular composition of the prostate microenvironment. PLoS ONE.

[176] Li X., Li Y., Dong L., Chang Y., Zhang X., Wang C., Chen M., Bo X., Chen H., Han W. (2023). Decitabine priming increases anti–PD-1 antitumor efficacy by promoting CD8+ progenitor exhausted T cell expansion in tumor models. J. Clin. Investig..

[177] Zhang H., Pang Y., Yi L., Wang X., Wei P., Wang H., Lin S. (2025). Epigenetic regulators combined with tumour immunotherapy: Current status and perspectives. Clin. Epigenet..

[178] Hu X., Lai W., Liu M., Wang B. (2026). Epigenetic dynamics of cellular senescence: From mechanistic insights to precision senotherapy. Chin. Med. J..

[179] Bouchlaka M.N., Sckisel G.D., Chen M., Mirsoian A., Zamora A.E., Maverakis E., Wilkins D.E., Alderson K.L., Hsiao H.-H., Weiss J.M. (2013). Aging predisposes to acute inflammatory induced pathology after tumor immunotherapy. J. Exp. Med..

[180] Garcia M.G., Deng Y., Murray C., Reyes R.M., Padron A., Bai H., Kancharla A., Gupta H., Shen-Orr S., Curiel T.J. (2022). Immune checkpoint expression and relationships to anti-PD-L1 immune checkpoint blockade cancer immunotherapy efficacy in aged versus young mice. Aging Cancer.

[181] Bailén R., Iacoboni G., Delgado J., López-Corral L., Hernani-Morales R., Ortiz-Maldonado V., Guerreiro M., Caballero A.C., Guerra-Domínguez M.L., Sánchez-Pina J.M. (2024). Anti-CD19 CAR-T cell therapy in elderly patients: Multicentric real-world experience from GETH-TC/GELTAMO. Transpl. Cell. Ther..

[182] Zak J., Pratumchai I., Marro B.S., Marquardt K.L., Zavareh R.B., Lairson L.L., Oldstone M.B., Varner J.A., Hegerova L., Cao Q. (2024). JAK inhibition enhances checkpoint blockade immunotherapy in patients with Hodgkin lymphoma. Science.

[183] Huang E., Ma T., Zhou J., Ma N., Yang W., Liu C., Hou Z., Chen S., de Castria T.B., Zeng B. (2022). The development and validation of a novel senescence-related long-chain non-coding RNA (lncRNA) signature that predicts prognosis and the tumor microenvironment of patients with hepatocellular carcinoma. Ann. Transl. Med..

[184] Liu Z., Ren C., Cai J., Yin B., Yuan J., Ding R., Ming W., Sun Y., Li Y. (2023). A novel aging-related prognostic lncRNA signature correlated with immune cell infiltration and response to immunotherapy in breast cancer. Molecules.

[185] Shi J., Hou Z., Fan L., Hu C., Ma N., Huang E. (2024). Development and experimental validation of a senescence-related long non-coding RNA signature for prognostic prediction and immune microenvironment characterization in gastric cancer patients. J. Gastrointest. Oncol..

